# Trimeric, APC-Targeted Subunit Vaccines Protect Mice against Seasonal and Pandemic Influenza

**DOI:** 10.1128/jvi.01694-22

**Published:** 2023-01-31

**Authors:** Elias Tjärnhage, Diamond Brown, Bjarne Bogen, Tor Kristian Andersen, Gunnveig Grødeland

**Affiliations:** a Institute of Clinical Medicine, University of Oslo, Oslo, Norway; b Department of Immunology, Oslo University Hospital, Oslo, Norway; The Peter Doherty Institute for Infection and Immunity

**Keywords:** DNA vaccines, adaptive immunity, influenza, influenza vaccines, subunit vaccine

## Abstract

Viral subunit vaccines contain the specific antigen deemed most important for development of protective immune responses. Typically, the chosen antigen is a surface protein involved in cellular entry of the virus, and neutralizing antibodies may prevent this. For influenza, hemagglutinin (HA) is thus a preferred antigen. However, the natural trimeric form of HA is often not considered during subunit vaccine development. Here, we have designed a vaccine format that maintains the trimeric HA conformation while targeting antigen toward major histocompatibility complex class II (MHCII) molecules or chemokine receptors on antigen-presenting cells (APC) for enhanced immunogenicity. Results demonstrated that a single DNA vaccination induced strong antibody and T-cell responses in mice. Importantly, a single DNA vaccination also protected mice from lethal challenges with influenza viruses H1N1 and H5N1. To further evaluate the versatility of the format, we developed MHCII-targeted HA from influenza A/California/04/2009(H1N1) as a protein vaccine and benchmarked this against Pandemrix and Flublok. These vaccine formats are different, but similar immune responses obtained with lower vaccine doses indicated that the MHCII-targeted subunit vaccine has an immunogenicity and efficacy that warrants progression to larger animals and humans.

**IMPORTANCE** Subunit vaccines present only selected viral proteins to the immune system and allow for safe and easy production. Here, we have developed a novel vaccine where influenza hemagglutinin is presented in the natural trimeric form and then steered toward antigen-presenting cells for increased immunogenicity. We demonstrate efficient induction of antibodies and T-cell responses, and demonstrate that the vaccine format can protect mice against influenza subtypes H1N1, H5N1, and H7N1.

## INTRODUCTION

Hemagglutinin (HA) is the major integral surface glycoprotein of influenza viruses and extracellularly consists of a stem domain and a globular head domain. It is trimeric in its natural state and binds sialic acid (SA) moieties on host cells (1). Binding to SA leads to internalization of the virus through endocytosis, which triggers a conformational change of HA that causes fusion between viral and endosomal membranes and thereby release of the viral genome (2). The SA binding site on the highly immunogenic globular head contains important epitopes to which neutralizing antibodies can bind, potentially mediating sterilizing immunity by blocking the virus from binding host cells (3–6).

Currently used influenza vaccines base their efficacy on neutralizing antibodies against HA (7, 8). Due to antigenic drift, a prolonged production time increases the probability of mismatches between vaccine-inserted strains and the viral strains circulating to cause seasonal epidemics in the population (9–11). Subunit vaccines improve production speed compared to conventional virus-containing vaccines, but typically have a lower immunogenicity (12). Further, novel HA-based vaccines often use monomeric HA (13–17), which could result in the loss of interface conformational epitopes (18). That said, a soluble HA ectodomain has previously been stabilized by the addition of a trimerization peptide from the T4 phage fibrillin protein, also known as foldon domain (19), leading to increased immunogenicity (20, 21).

Targeting of antigens to surface markers and receptors on antigen-presenting cells (APC) has been shown to greatly enhance antigen immunogenicity (22–27), and we have previously demonstrated that DNA vaccines encoding APC-targeted HA raised protective antibody levels in mice and larger animals (13, 28–31). More specifically, we demonstrated that targeting of HA to major histocompatibility class II (MHCII) molecules was particularly efficient at raising antibody responses, whereas targeting to chemokine receptors (CCR) 1 and 5 induced a more cellular-based immune response (28). These vaccines were designed with an X-shaped structure, with two arms containing APC-specific targeting moieties and two arms containing monomeric HA (13). The resulting bivalent antigen display was likely important for cross-linking of B-cell receptors, and as such, also activation of immune responses (32). However, the bivalent display of monomeric HA did not take into consideration the possibility that important B-cell epitopes could be located at the interphases between monomers in a trimeric conformation.

Here, we have designed new APC-targeted vaccines with the aim to enable display of HA in its natural trimeric state. First, HA was linked directly to an APC-specific targeting moiety, as such omitting the previously used dimerization unit that led to bivalent display in an X-shaped structure. Second, we constructed vaccines with HA linked to either the chemokine macrophage inflammatory protein 1 alpha (MIP1α) or a single-chain variable fragment (scFv) specific for MHCII molecules, as these were previously demonstrated to be favorable for protection against influenza when comparing nine different APC-specific targeting moieties (33). Third, we designed vaccines with or without a foldon trimerization domain at the C terminus to see whether this influenced antigen conformation and immunogenicity. In sum, we designed APC-targeted vaccines where selective targeting to MHCII or chemokine receptors could improve vaccine efficacy while also retaining the native trimeric HA structure to potentially induce the wider antibody repertoire associated with live infections (34).

## RESULTS

### Construction and structure of APC-targeted HA proteins.

**FIG 1:**
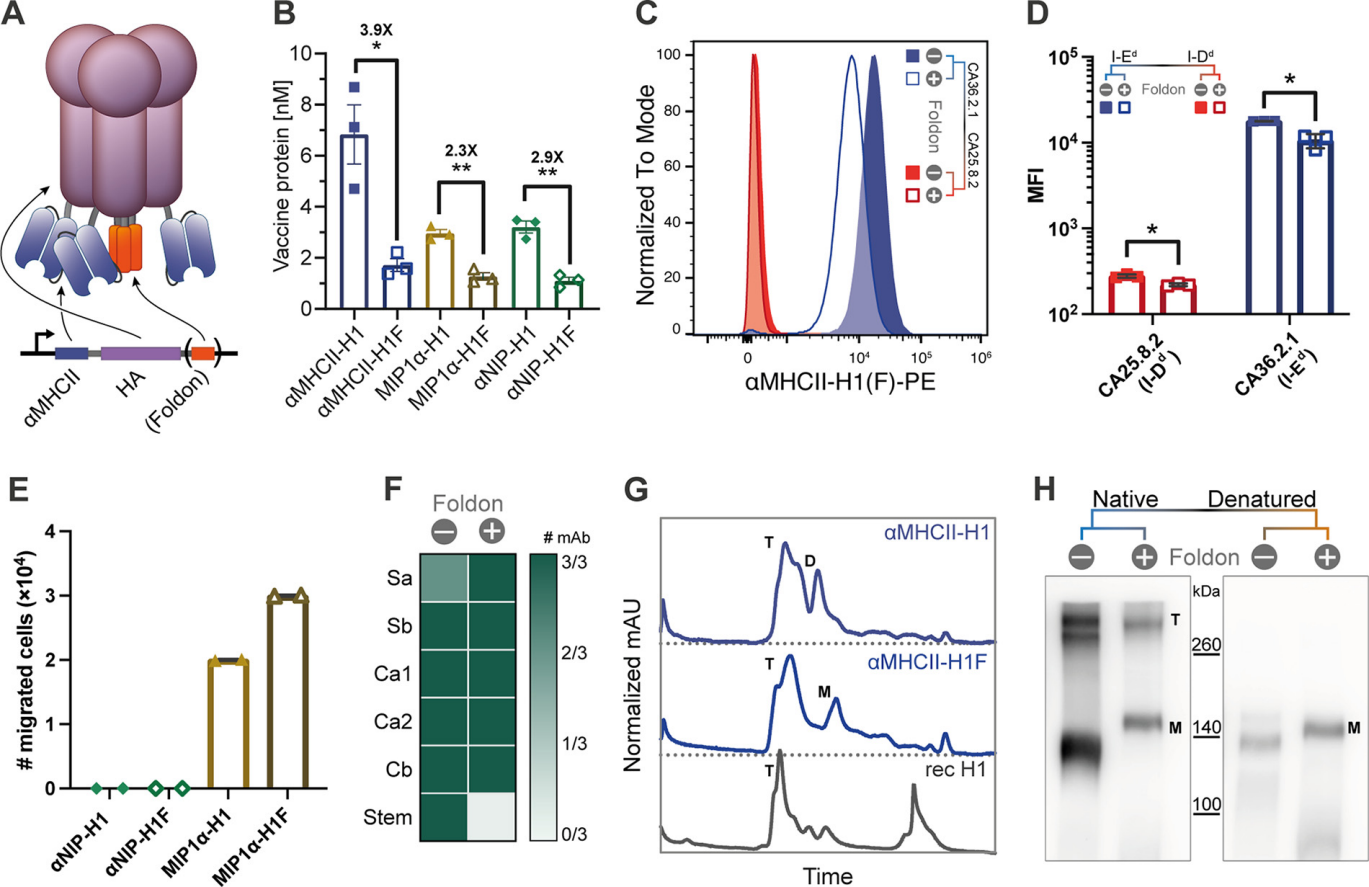
Vaccine protein secretion and structure. (A) Schematic representation of the genetic vaccine and the produced protein, here represented by αMHCII-H1F. (B) ELISA of supernatants from transiently transfected HEK293E cells. Bars indicate the mean ± standard error of mean (SEM) with the fold increase shown for significant increases (two-sided Mann-Whitney test). (C) Binding of MHCII-targeted vaccines to MHCII (E^d^)-expressing cells (CA36.2.1), but not to MHCII (D^d^)-expressing cells (CA25.8.2). (D) Mean fluorescence intensity of data from panel C (mean ± SD). (E) Chemotaxis of cells after stimulation with MIP1α-containing vaccines compared to nontargeted controls (two independent experiments, mean ± SD). (F) Conformational epitope mapping of secreted vaccine proteins by sandwich ELISA with a panel of monoclonal antibodies with known binding epitopes on HA (Sa, Sb, Ca1, Ca2, Cb, and stem). Three MAbs were used per epitope (36). (G) Size exclusion chromatography of purified vaccine protein compared to commercially available HA. Peaks corresponding to trimers, dimers, and monomers are indicated by T, D, and M, respectively. (H) Western blot analysis of purified vaccine proteins under native and denaturing conditions, detected by MAb against HA(PR8) (clone: H36-4-52). Bands corresponding to trimers, dimers, and monomers are indicated by T, D, and M, respectively.

The vaccines were genetically constructed by adding an APC-specific targeting domain encoding either an scFv specific for murine major histocompatibility complex class II (MHCII) molecules (I-E^d^) (27), or the chemokine murine macrophage inflammatory protein 1 alpha (MIP1α) (35), to the N terminus of HA, separated by a linker (GESYAEAAAKEAAAK) (Fig. 1A). A nontargeted control vaccine was prepared by replacing the scFv specific for MHCII with a scFv against the hapten 4-hydroxy-3-iodo-5-nitrophenylacetic (NIP) (27). The vaccines were then either genetically linked or not to a C-terminal trimerization domain (19) (see Table S1 in the supplemental material). The theoretical size for the vaccine monomers composed of one HA monomer (~60 kDa) plus one scFv (~26 kDa) with linkers is about 90 kDa, and with an added 25 to 30 kDa worth of glycan moieties (Fig. S1A), yielding a total vaccine monomer size of around 120 kDa.

In order to evaluate protein expression, the constructed DNA vaccines were transiently transfected into HEK293E cells. Vaccine proteins with HA from influenza A/PR/8/1934 (H1N1) (here denoted H1) as antigen were secreted in the range of 100 to 400 ng/mL (Fig. 1B). Vaccines without the C-terminal trimerization domain were expressed at significantly higher protein levels compared to the vaccines with this domain (denoted H1F). Cells transfected with αMHCII-H1 expressed the highest levels of vaccine proteins, while MIP1α-H1 and αNIP-H1 were expressed at equal and lower levels.

The efficacy of the APC-targeted vaccines is dependent on the functionality of the APC-specific targeting moiety (13, 27, 28, 35). Thus, we evaluated binding of MHCII-targeted vaccines (I-E^d^ specific) to cells expressing relevant E^d^ or irrelevant D^d^ molecules. Importantly, the vaccines efficiently bound cells expressing E^d^, but not D^d^ (Fig. 1C and D). For vaccines targeting chemokine receptors via the chemokine MIP1α, chemotactic integrity of the targeting moiety was confirmed by evaluating cellular migration across a membrane in response to the titrated presence of vaccine proteins (Fig. 1E).

The expressed vaccine proteins were next screened against a panel of 18 monoclonal antibodies (MAbs) with known binding toward established HA epitopes in order to evaluate antigen folding to more detail (21, 36) (Fig. 1F). Importantly, 3/3 MAbs against the known immunodominant sites Sb, Ca1, Ca2, and Cb bound vaccine proteins regardless of the presence of a trimerization domain, and the Sa site was recognized by 2/3 MAbs when foldon was left out and by 3/3 when it was included. In addition, 3/3 stem binding MAbs recognized the HA stem in the vaccine without the trimerization domain, while 0/3 clones recognized the corresponding vaccine with the trimerization domain. Interestingly, looking at the fold change in signal for vaccine- over mock-transfects revealed that certain epitopes may be favored by the different vaccines. As an example, foldon vaccines displayed a higher fold change signal for the Sa site in all three MAbs, whereas the vaccines without a foldon had a higher signal for the Sb site in 2/3 MAbs (Fig. S1B).

To further evaluate the impact of a trimerization domain, analytical size exclusion and Western blotting of purified vaccine proteins were performed. When comparing the sizes of the vaccines with commercially available recombinant HA protein and standards of known size on a Superdex 200 Increase 3.2/300 high-pressure liquid chromatography (HPLC) size exclusion column, we found that a majority of the proteins were trimeric, with some possible breakdown products at lower molecular weights. Based on areas under the peak, an estimated 80 to 85% of the protein eluted in the trimeric peak for both vaccine formats (Fig. 1G). The observation that the produced vaccine proteins were dominantly trimeric independently of a trimerization domain was confirmed also by Western blotting (Fig. 1H). Trimeric vaccines were seen under nondenaturing conditions, but not under denaturing conditions. Some monomeric vaccines were observed under nondenaturing conditions as well, indicating that the trimeric vaccines may not be very stable.

### *In vivo* humoral immune responses.

**FIG 2:**
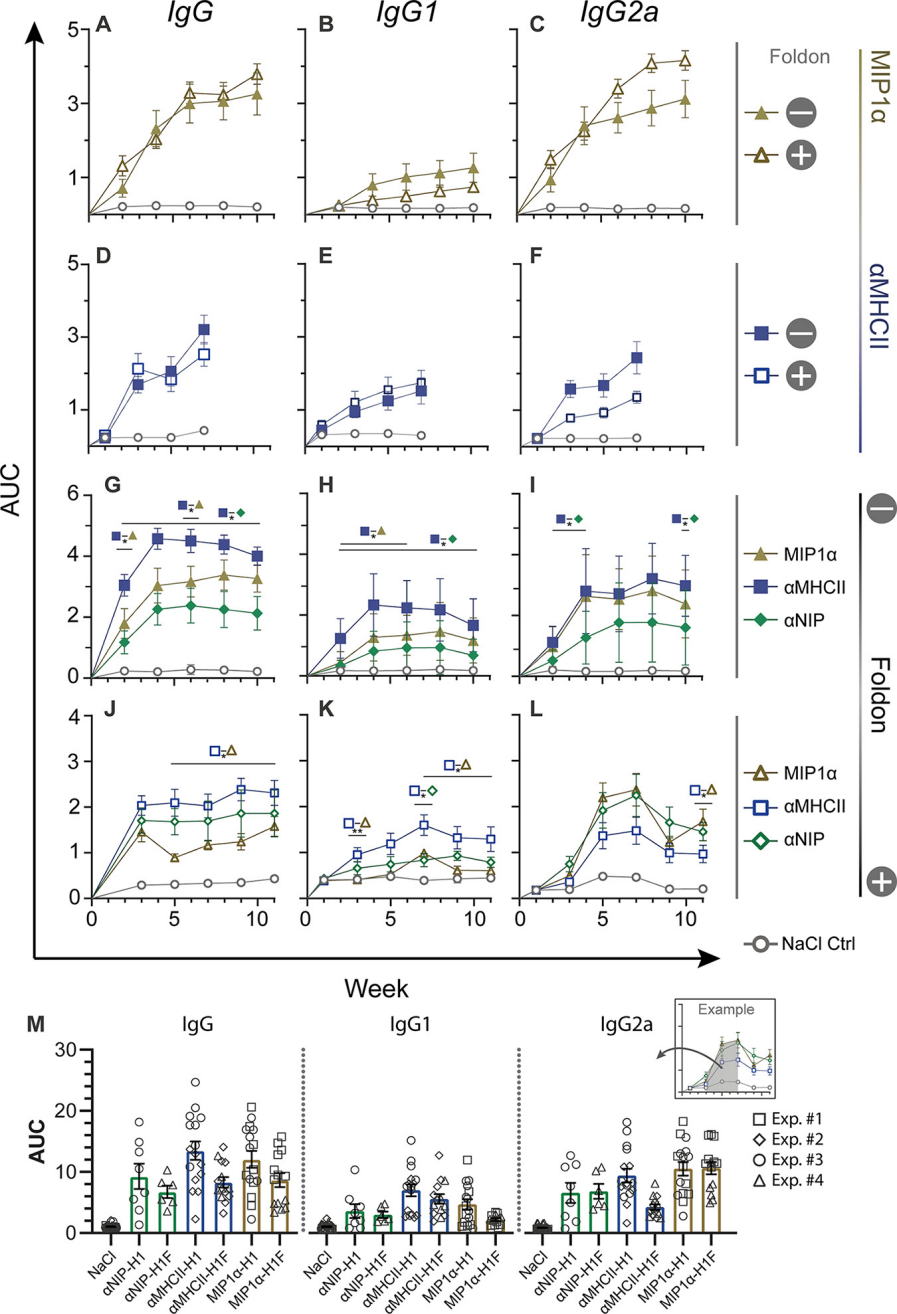
Efficient induction of antibodies in sera after DNA vaccination. Mice were immunized with 50 μg plasmid i.m., and blood samples were collected every 2 weeks (*n* = 8 mice/group for panels A to I; *n* = 6 mice/group for panels J to L). Serum antibody responses measured by ELISA, shown as AUC over time. (Left column) Total IgG responses. (Middle column) IgG1 responses. (Right column) IgG2a responses. (A to C) Comparison of MIP1α-H1 and MIP1α-H1F. (D to F) Comparison of αMHCII-H1 and αMHCII-H1F. (G to I) Comparison of differently targeted vaccines without trimerization domain. (J to L) Comparison of differently targeted vaccines with trimerization domain. (M) All serum IgG responses in panels A to L. Compiled responses were calculated based on the AUC from weeks 2 to 8. Different symbols indicate to which experiment the specific data points belong. Statistical analysis was performed by pairwise Mann-Whitney test at each time point. *, *P* < 0.05; **, *P* < 0.005. Data are displayed as the mean ± SEM.

To assess vaccine immunogenicity *in vivo*, BALB/c mice were vaccinated intramuscularly (i.m.) with plasmids encoding the different vaccines, followed by electroporation of the injection site to facilitate cellular DNA uptake. Antibody responses were assessed longitudinally in sera from 2 weeks after immunization, with the levels generally increasing over 10 weeks (Fig. 2). Interestingly, we did not observe any significant differences in total HA-specific serum IgG, IgG1, and IgG2a for vaccines with or without the trimerization domain (Fig. 2A to F).

Experiments were next set up to more directly compare the contribution of the different APC-specific targeting moieties in the absence or presence of a trimerization domain (Fig. 2G to L). Results demonstrated significantly higher serum levels of total IgG and IgG1 after vaccination with αMHCII-H1 compared to the nontargeted control vaccine αNIP-H1 (Fig. 2G to I). αMHCII-H1 also raised significantly higher IgG2a responses than αNIP-H1 at weeks 2 to 4 and 10 after vaccination. For MIP1α-H1 versus αNIP-H1, the difference was not significant for total IgG, IgG1, or IgG2a, even though the mean of the serum IgG responses was higher for MIP1α-H1 at all time points.

When the trimerization domain was included, many of the significant differences between targeted and nontargeted vaccines disappeared (Fig. 2J to L). However, the mean IgG and IgG1 responses after vaccination with αMHCII-H1F were consistently higher than the responses after vaccination with αNIP-H1F. When comparing MHCII-targeted to CCR1/5-targeted vaccines, we observed that MHCII-targeted vaccines both with and without a trimerization domain induced higher total IgG and IgG1 responses. This was reversed for IgG2a, where responses for MIP1α-H1/H1F and αMHCII-H1/H1F were more similar.

When summing up the four different experiments, we observed that IgG, IgG1, and IgG2a responses were higher in vaccines without a foldon, and that the APC-targeted vaccines mostly improved responses over the nontargeted control vaccine αNIP-H1/H1F (Fig. 2M). Further, a polarization toward IgG and IgG1 for MHCII-targeted vaccines and IgG2a for CCR-targeted vaccines was observed, which is in accordance with previous observations (28, 29).

### *In vivo* cellular immune responses.

**FIG 3:**
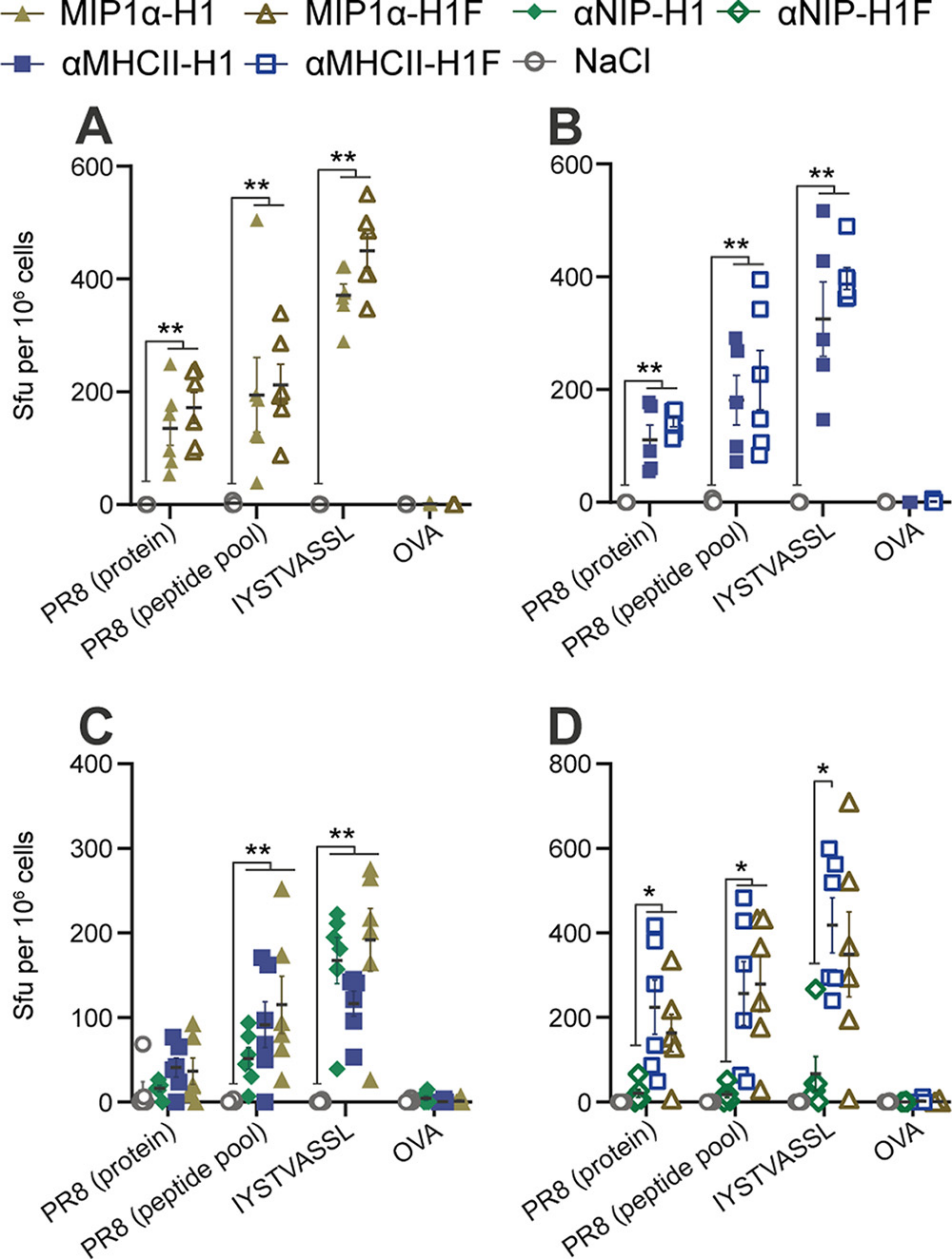
Vaccine-induced cellular immunity. Splenocytes from 14 days postimmunization were stimulated *in vitro* with rec. HA from influenza PR8, OVA, an HA (PR8) overlapping peptide pool, or the class I-restricted peptide IYSTVASSL, and assayed for IFN-γ secretion by ELISpot (*n* = 6 mice/group; each shown point is the mean of 3 technical replicates per mouse). Bars indicate the mean ± SEM. (A and B) Comparison with or without trimerization domain for (A) MIP1α vaccines and (B) MHCII-targeted vaccines. (C and D) Comparison of different APC-specific targeting moieties (C) without trimerization domain and (D) with trimerization domain. Statistical analyses were performed by pairwise comparisons of each group using two-sided Mann-Whitney tests, *, *P* < 0.05; **, *P* < 0.005. Data are displayed as the mean ± SEM.

Similar to antibody responses, T-cell responses will contribute to the formation of protective immunity in vaccinees. Thus, we stimulated splenocytes from vaccinated mice with H1 protein and a peptide pool spanning H1, as well as the MHCI (H-2K^d^) restricted HA peptide IYSTVASSL, in an ELISpot assay evaluating gamma interferon (IFN-γ) secretion. Importantly, both the APC-targeted vaccines with and without a trimerization domain could significantly raise IFN-γ secretion above that of the saline control (Fig. 3A and B), and we observed no significant influence from the presence of a trimerization domain in the APC-targeted vaccines.

Next, we set up experiments to evaluate the efficacy of APC-targeting more directly (Fig. 3C and D). Results demonstrated that αMHCII-H1, MIP1α-H1, and αNIP-H1 all significantly raised IFN-γ secretion above that of the saline control (Fig. 3C). In contrast, both the APC-targeted vaccines with a foldon significantly raised the levels of IFN-γ-producing cells compared to αNIP-H1F (Fig. 3D). In sum, we observed that APC-targeting of antigen enhanced formation of cellular immunity after vaccination in the presence of a trimerization domain, and that this effect was reduced in its absence.

### APC-targeted HA protects against lethal viral challenge.

**FIG 4:**
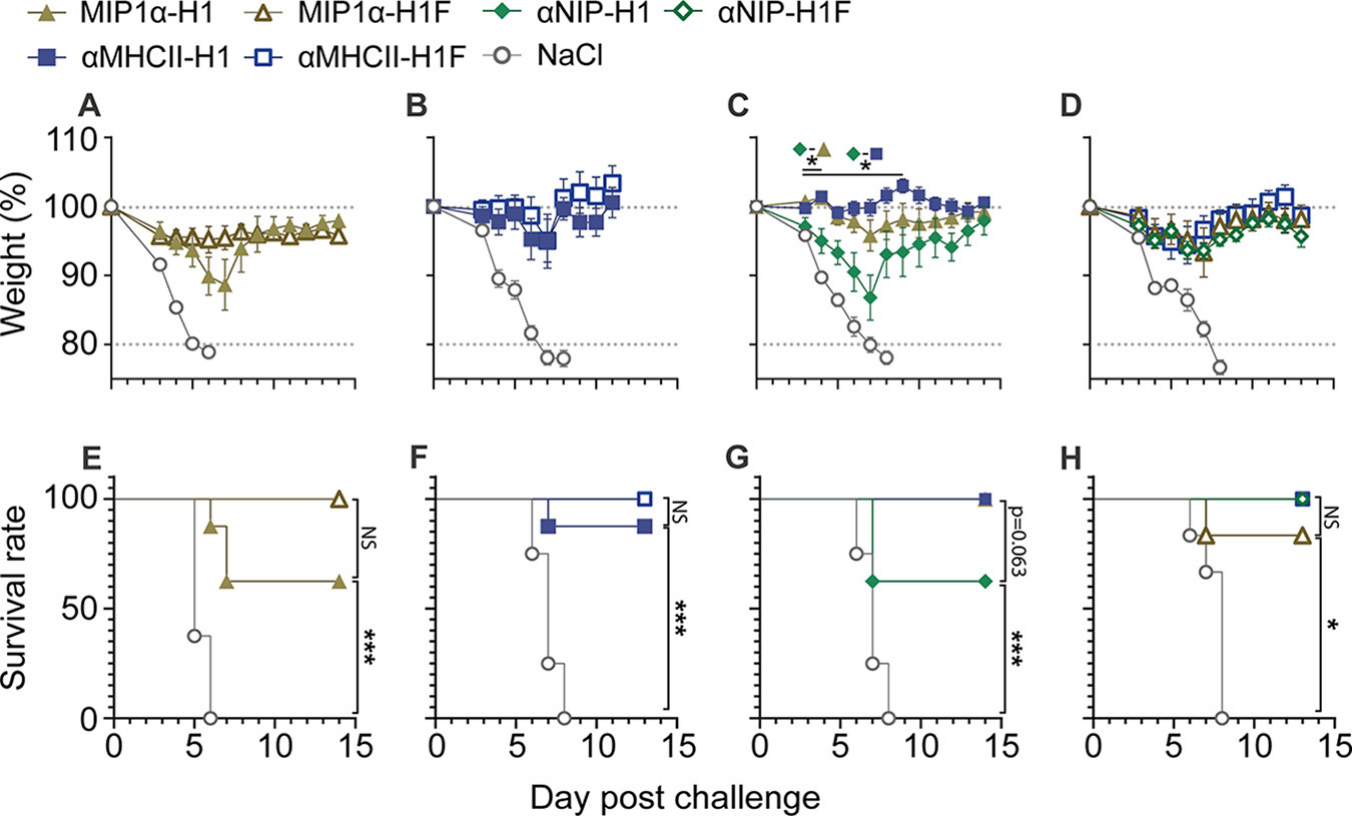
Vaccine effectiveness against a lethal influenza challenge. Mice were vaccinated with a single dose of the indicated vaccines and challenged 7 to –13 weeks postvaccination with a 5 × LD_50_ dose of influenza PR8 intranasally (i.n.) (*n* = 8 [panels A to C and E to G] or 6 [panels D and H] mice/group). Mice were monitored daily for weight loss; data are displayed as the weight mean ± SEM (panels A to D), with a humane endpoint of 20% body weight loss as basis for survival curves (panels E to H). (A and B) Comparison of vaccines with or without a trimerization domain: (A) weight after PR8 challenge 10 weeks postvaccination with MIP1α vaccines and (B) weight after challenge 7 weeks postvaccination with MHCII-targeted vaccines. (C and D) Comparison of different APC-specific targeting moieties: weight following viral challenge is shown for vaccines either (C) without a trimerization domain 10 weeks after vaccination or (D) with a trimerization domain 13 weeks after vaccination. Significant weight loss was determined by group-wise comparison using a two-sided Mann-Whitney test for each time point. Significance between vaccine groups is shown above the corresponding time point; *, *P* < 0.05; **, *P* < 0.005. (E to H) Survival curves corresponding to the above-described weight panels. Significance was calculated by log-rank (Mantel-Cox) tests. *, *P* < 0.0332; **, *P* < 0.0021; ***, *P* < 0.0002.

Key to vaccine functionality is its ability to protect against disease. Thus, we challenged vaccinated mice with a lethal dose of influenza A/Puerto Rico/8/1934(H1N1) (PR8) and monitored for weight as a marker of disease (Fig. 4). In accordance with the above-described data, we did not observe a significant difference in weight between groups receiving APC-targeted vaccines with or without the trimerization domain (Fig. 4A and B). When evaluating the effect of APC-targeting, however, we observed significant differences in weight loss between the nontargeted control vaccine αNIP-H1 and αMHCII-H1 from days 3 to 9 and between αNIP-H1 and MIP1α on days 3 to 4 (Fig. 4C). This difference was lost when the trimerization domain was included (Fig. 4D). This same trend was also observed when evaluating survival curves based on 20% weight loss as the humane endpoint (Fig. 4E to H).

### Trivalent APC-targeted HA vaccination.

There are 18 subtypes of influenza A that can be classified based on differences in HA. Seasonal influenza is presently caused by H1N1 or H3N2 influenza viruses. Accordingly, conventional influenza vaccines contain a selected strain from these two subtypes, selected based on the expectation that they will be relevant for preventing next season’s epidemic. In addition to seasonal epidemics, there is a real potential that influenza subtypes currently circulating in birds may reassort to variants able to transmit among humans. Thus, we constructed MHCII- and CCR-targeted vaccines encoding HA from A/Vietnam/1194/2004(H5N1) (VN04) and A/Hong Kong/1073/99(H9N2) (HK99) to represent subtypes with a high potential for future zoonosis. We chose to prepare only vaccines without added trimerization domains since we did not observe significant differences between vaccines equipped with a foldon or not in the previous experiments (Fig. 2A to F, Fig. 3A and B, and Fig. 4A and B). Following transient transfection of the different vaccine plasmids in cell culture, efficient secretion of vaccine proteins with HA from H1, H5, and H9 influenza viruses was confirmed by enzyme-linked immunosorbent assay (ELISA) (Fig. S2).

**FIG 5:**
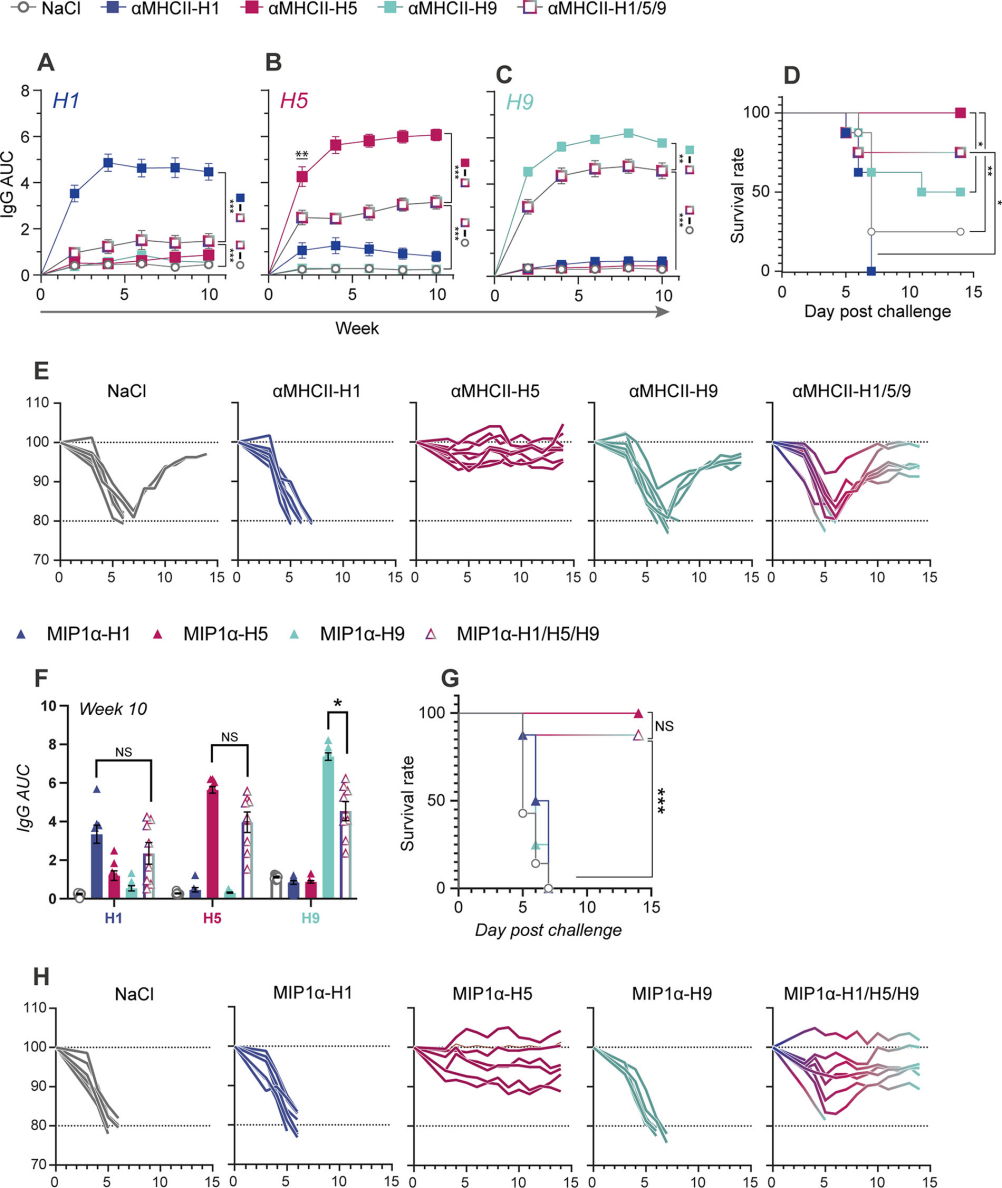
Efficient induction of antibodies and protection against both seasonal (H1) and potentially pandemic influenza subtypes (H5, H9). Mice were immunized i.m. with 45 μg DNA per mouse, either a mixture of 15 μg DNA per HA in trivalent mix or 45 μg of the indicated monovalent vaccines delivered independently (*n* = 8 per group, mean ± SEM). (A to E) MHCII-targeted monovalent and trivalent vaccines. Longitudinal serum IgG responses in ELISA against HA from influenza (A) H1 (PR8), (B) H5 (VN04), and (C) H9 (HK99), displayed as AUC. Statistical analysis was performed by a pairwise Mann-Whitney test at each time point. Shown is the statistical comparison between the trivalent and corresponding monovalent vaccine and saline control. Data are displayed as the mean ± SEM. (D) Survival rates after a viral challenge with a 5 × LD_50_ dose of influenza H5N1 virus, defined by 20% weight loss. (E) Weight of individual mice corresponding to panel D. (F to H) CCR-targeted (with MIP1α) monovalent and trivalent vaccines. (F) Serum IgG responses on week 10 after vaccination, measured in ELISA against HA from the indicated viruses. Two-sided Mann-Whitney tests between the trivalent and corresponding monovalent vaccine are shown. Data are displayed as the mean ± SEM. (G) Survival rates after viral challenge 10 weeks postvaccination with 5 × LD_50_ influenza H5N1 virus, defined by humane endpoint of 20% weight loss. (H) Weight from individual mice corresponding to panel G. Mann-Whitney test significance: *, *P* < 0.05; **, *P* < 0.005; ***, *P* < 0.0005. Survival statistics were analyzed by log-rank (Mantel-Cox) and Gehan-Breslow-Wilcoxon tests: *, *P* < 0.0332; **, *P* < 0.0021; ***, *P* < 0.0002.

The MHCII-targeted vaccines encoding HA from influenza H5N1 and H9N2 were mixed with αMHCII-H1, and mice were vaccinated with either this mixture or the three vaccines independently. The total DNA concentration administered to each mouse was kept constant for the mixture and vaccination with a single vaccine. When evaluating antibody responses after vaccination, we correspondingly observed that vaccination with either αMHCII-H1, αMHCII-H5, or αMHCII-H9 significantly elevated responses above vaccination with the mixture (Fig. 5A to C). The vaccine-induced antibody responses were strain specific, except that αMHCII-H1 also raised some antibodies that cross-reacted with H5 (Fig. 5B). Vaccination with αMHCII-H1/5/9 raised significant IgG responses against HA from H1, H5, and H9 influenza viruses.

At 11 weeks after a single vaccination, mice were challenged with a lethal dose of influenza H5N1 (Fig. 5D and E). In accordance with the observed antibody responses, αMHCII-H5 offered complete protection against disease. In the group receiving the trivalent mixture, the mice initially lost weight, but 6/8 recovered from the infection. In contrast, 4/8 vaccinated with αMHCII-H9 recovered after a weight loss, and 2/8 in the saline control group recovered. All mice vaccinated with αMHCII-H1 succumbed to infection.

Next, we vaccinated mice with CCR-targeted vaccines against H1, H5, or H9 or a mixture thereof. Similar to the above-described MHCII-targeted vaccinations, DNA concentrations were kept constant for the different groups. As expected, vaccination with CCR-targeted vaccines displaying H1, H5, or H9 alone induced strong responses in an ELISA against homologous proteins (Fig. 5F). Interestingly, these responses were equaled in sera collected from mice vaccinated with the mix vaccine MIP1α-H1/H5/H9 when assayed against HA from influenza H1 and H5 viruses. Responses against H9 were significantly reduced for vaccination with MIP1a-H1/H5/H9 compared to MIP1a-H9, but still markedly present.

At 11 weeks postvaccination, mice were challenged with a lethal dose of influenza H5N1. In accordance with the observed antibody responses, 8/8 mice vaccinated with MIP1α-H5 survived, and 7/8 vaccinated with MIP1α-H1/5/9 survived (Fig. 5G and H).

### Protein-based APC-targeted HA subunit vaccine.

The first DNA vaccine for human use was recently approved for clinical use against SARS-CoV-2 in India (37), but even with the approved mRNA vaccines, there has been some public concern about the potential consequences of genomic integration. Thus, we wanted to examine how the APC-targeted HA subunit vaccine would work in a protein-based format. Here, HA from A/Puerto Rico/8/1934(H1N1) in αMHCII-H1 was replaced with HA from A/California/07/2009(H1N1) (CA07) (αMHCII-CA07). The MHCII-targeted vaccine was chosen here as our candidate vaccine since this format had proven more efficient at antibody induction (Fig. 2G and H), which is the protective mechanism for conventional vaccines. Thus, αMHCII-CA07 vaccine proteins were produced by transient transfection in 293E cells, and affinity purified for HA (Fig. S3A and B).

Mice were vaccinated with the αMHCII-CA07 vaccine proteins, with or without the adjuvant AS03. To enable benchmarking against commercially available vaccines, we also included groups vaccinated with Flublok (38) and Pandemrix (39). αMHCII-CA07 was given at 3.5 μg per mouse, whereas a dose of Flublok (quadrivalent, formulated with 2 influenza A and 2 influenza B subtypes) or Pandemrix (formulated with AS03) corresponding to 9 μg per HA was administered. Blood samples were taken at 2-week intervals for 12 weeks before a viral challenge with influenza CA07 virus.

**FIG 6:**
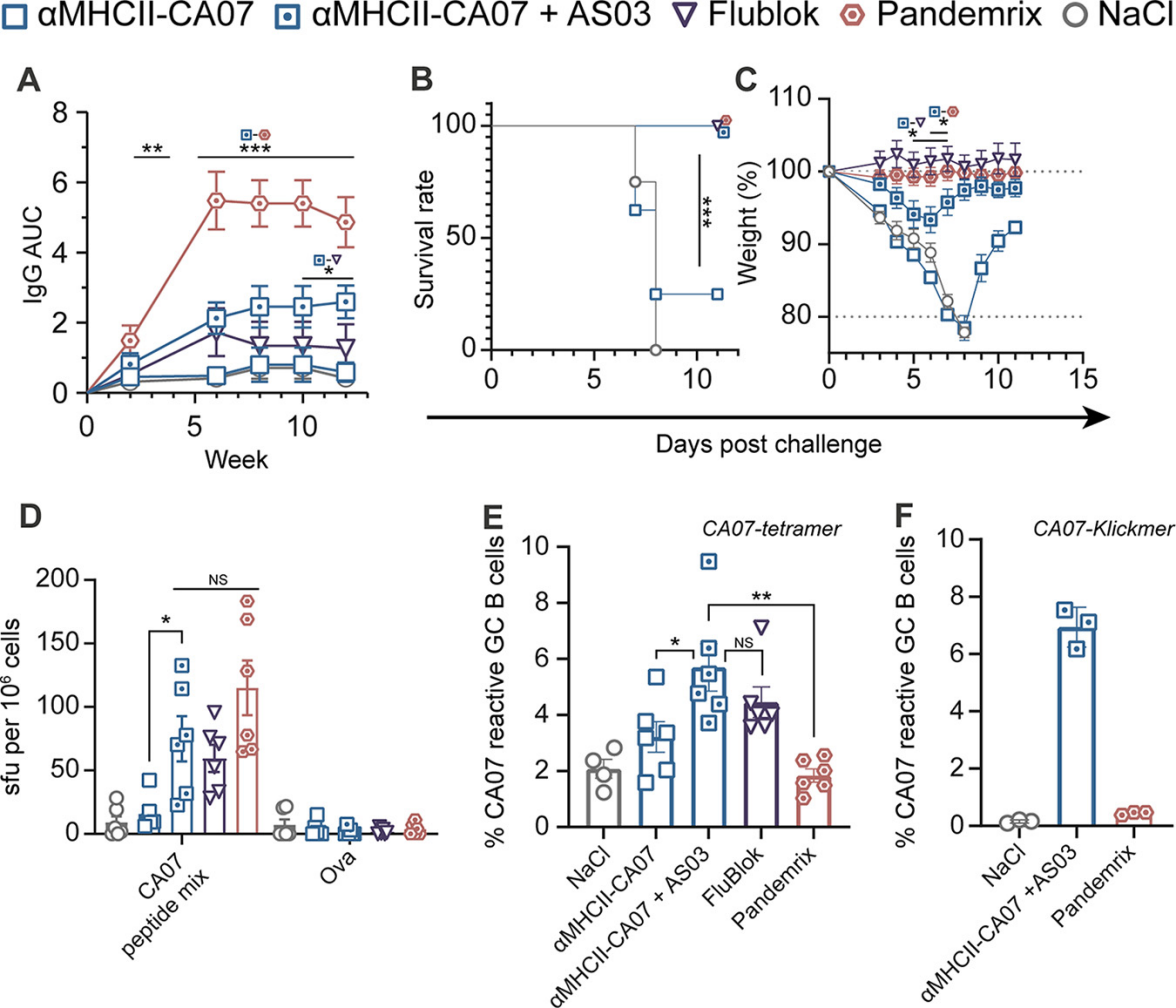
Protein vaccination with MHCII-CA07 induced B- and T-cell responses and protected against a lethal influenza challenge. (A to C) Mice were immunized with 9 μg HA protein per mouse for Flublok and Pandemrix or 3.5 μg of αMHCII-CA07. (A) Serum IgG levels assayed against CA07 HA in ELISA. Statistics were determined by a pairwise Mann-Whitney test at each time point. (B) Survival rate as defined by a humane endpoint of 20% weight loss; (C) weight after viral challenge with a 5 × LD50 dose of A/California/07/2009(H1N1) (Cal07) virus (*n* = 8 per group, mean ± SEM). The survival rate was analyzed by log-rank (Mantel-Cox) and Gehan-Breslow-Wilcoxon tests. (D to F) Mice were immunized with 9 μg CA07 HA per mouse for Pandemrix, Flublok, and αMHCII-CA07. (D) IFN-γ ELISpot of splenocytes collected on day 14 after a single immunization (*n* = 8 per group, mean ± SEM). (E and F) CA07-specific GC B cells on day 14 postimmunization, (E) with tetramer or (F) with Klickmer-based staining for flow cytometry. GC B cells were gated as single cells, TCRβ^neg^, CD19^pos^, B220^pos^, CD38^neg^, and GL7^int-hi^ (*n* = 6 per group, individual replicates ± SD). Statistical analysis by Mann-Whitney tests: *, *P* < 0.05; **, *P* < 0.005; ***, *P* < 0.0005. Survival statistics were analyzed by log-rank (Mantel-Cox) and Gehan-Breslow-Wilcoxon tests: *, *P* < 0.0332; **, *P* < 0.0021; ***, *P* < 0.0002.

Vaccination with the split virus vaccine Pandemrix+AS03 quickly raised antibody titers, and responses were significantly elevated above those observed for the other vaccine formats (Fig. 6A). The AS03 adjuvanted αMHCII-CA07 proteins raised total IgG responses to a similar level as Flublok initially, but significantly higher than Flublok from week 8. Nonadjuvanted αMHCII-CA07 did not induce significant levels of serum IgG above that of the saline control (Fig. 6A).

For an assessment of the protective potential of the different vaccines, mice were challenged with a lethal dose of influenza virus A/California/07/2009(H1N1). Importantly, there were no significant differences in survival observed for αMHCII-CA07+AS03, Flublok, and Pandemrix+AS03 (Fig. 6B), but a small and transient weight loss was observed at days 3 to 5 following vaccination with αMHCII-CA07+AS03 (Fig. 6C).

To complement the picture of immune formation after vaccination with the different protein or virus vaccines, we wanted to evaluate cellular immune responses. Mice were vaccinated with 9 μg HA for all vaccines, and IFN-γ production was evaluated in splenocytes collected at day 14 postvaccination (Fig. 6D). Pandemrix+AS03, Flublok, and αMHCII-CA07+AS03 raised significantly higher numbers of IFN-γ-secreting cells than nonadjuvanted αMHCII-CA07 and the saline control group when stimulated with CA07 peptides but were not significantly different from each other.

Next, we wanted to examine germinal center (GC) B cells after vaccination by fluorescence-activated cell sorter (FACS) staining of draining lymph nodes. Antigen-specific GC B cells were identified by binding of HA-streptavidin tetramer probes. Interestingly, while vaccination with Pandemrix+AS03 raised the total number of GC B cells to exceed that induced by the other vaccines, vaccination with αMHCII-CA07+AS03 significantly elevated the number of HA-reactive GC B cells compared to Pandemrix+AS03 (Fig. 6E, Fig. S4A and B). A repeat of this experiment with HA-coupled Klickmer to enable improved selection of antigen-specific B cells confirmed this result (Fig. 6F, Fig. S4C).

In sum, we demonstrated that vaccination with a low dose of the protein version of αMHCII-CA07 could raise immune responses and protection comparably to that observed after vaccination with higher protein doses of commercially available influenza vaccines.

## DISCUSSION

Here, we have demonstrated that steering of trimerized HA to APC may enhance immune responses, including serum IgG (Fig. 2G to I), cellular immunity (Fig. 3D), and protection against a lethal influenza challenge (Fig. 4C and G). Interestingly, the vaccine tended to be secreted as a trimer whether a trimerization unit was present or not. We showed that the vaccine can be delivered in the form of both DNA vaccine and protein.

The natural state of HA is a trimeric conformation. Even though many of the well-characterized epitopes on HA are confined to a monomer, some, such as Ca1, span two monomers (36, 40). It is therefore interesting to develop vaccines that present trimeric HA to the immune system. Previously, a stabilized trimeric HA structure has also been shown to be more immunogenic (20, 21). While the addition of a foldon in our study did not significantly influence the proportion of trimeric vaccine proteins (Fig. 1G), the presence of a trimerization domain nevertheless influenced vaccine immunogenicity. More specifically, the difference between αMHCII-H1F and the nontargeted control vaccine αNIP-H1F was reduced in its presence (Fig. 2 and 4).

Previously, we have demonstrated that targeting of antigen to APC enhanced immune responses after a single DNA vaccination in mice and larger animals (13, 27, 31). The vaccine format used for these studies were based on bivalent monomeric display of antigens that were linked to an APC-specific targeting unit via a dimerization unit that structured monomeric antigens as two flexible arms of an X (13, 27). The dual-antigen display likely enabled cross-linking of B cell receptors (BCR) in an APC-B cell synapse, and we have correspondingly demonstrated bivalent antigen display favorable to monomeric display (32). For the present study, we omitted the previously used dimerization domain and found that HA could trimerize even in the absence of a foldon. The implication is trivalent display also of B cell epitopes, and that likely will facilitate cross-linking of BCR and efficient immune activation (41, 42).

Interestingly, we observed that the presence of a trimerization unit reduced protein secretion from transfected cells (Fig. 1B). This could be due to more rapid processing of newly produced vaccine protein in the absence of this addition, causing the potential enhanced stability not to play a key role. Protein expression *in vivo* is key for the efficacy of genetic vaccines, presenting an argument for progression toward clinical use with APC-targeted HA without a foldon. Further, the APC-specific targeting moiety is located at the N terminus of HA, which likely ends up next to the trimerization domain in the C terminus after HA folding (Fig. 1A). As such, steric interference could potentially explain both the reduced protein secretion observed and the failure of stem-specific antibodies to bind the vaccine equipped with the trimerization domain (Fig. 1F). Of note, we have previously observed that stem-binding antibodies could bind monomers (43), so binding should not be interpreted as confirmation of trimerization.

The flexibility of the subunit vaccine formats, such as DNA, mRNA, and protein, enable conscious engineering of the included antigens (44) or new combinations to produce the desired immune response. The influenza vaccines currently in use combine 3 to 4 strains or HA antigens from seasonal influenza A and B subtypes, while we here instead wanted to evaluate the combination of seasonal (H1) and potentially pandemic influenza subtypes (H5, H9). Importantly, the serum IgG responses observed against HA from influenza subtypes H5 and H9 in the trivalent mixture indicated a potential for these vaccines against potentially pandemic outbreaks of new influenza strains. At present, neither H5 nor H9 has evolved into a viral variant able to transmit between humans. However, annual zoonosis demonstrates mortality rates in the range of 50 to 60% for H5 (45, 46). It is imperative to have available vaccine strategies able to offer rapid protection against emerging H5 and H9 variants with a pandemic potential.

RNA and DNA vaccines have the advantage of enabling rapid production compared to conventional vaccines. However, no genetic vaccines were approved for use against viral infections until the recent SARS-CoV-2 pandemic, where the mRNA vaccines from Moderna (Spikevax) (47) and Pfizer-BioNTech (Comirnaty) (48) and the DNA vaccine from Zydus Cadila (ZyCoV-D) (49) were approved (37, 50, 51). While the vaccines have been demonstrated to be safe and efficient (52), the public has in some instances found it difficult to understand genetic vaccines, as they are perceived to potentially also be able to cause genomic integration (53, 54). A protein formulation of the vaccine may thus be easier to deploy in a large population, and enable more direct control over the administered dose of the antigen.

We compared a protein version of αMHCII-H1 to the commercially available vaccines Flublok and Pandemrix. Similar to αMHCII-CA07, Flublok is a recombinant HA subunit vaccine but is formulated as a quadrivalent mixture of two influenza A strains and two influenza B strains. Pandemrix is a split virion vaccine and is formulated as a monovalent vaccine since it was designed as a pandemic vaccine against the H1N1 pandemic of 2009. It is codelivered with the strong adjuvant AS03 (55). As such, a comparison of these three vaccine formats is a bit like comparing apples to pears. In addition, we used different doses of HA for the different vaccine formats; the commercial vaccines were given as 10% of the human dose, as is often done in mice, but we first used a lower dose of the MHCII-targeted protein vaccine. Nevertheless, benchmarking the responses observed after vaccination with αMHCII-CA07 to known vaccine types is of relevance. Pandemrix indeed raised stronger antibody responses than αMHCII-CA07, but the reduced responses may be favorable for a seasonal vaccine mostly aiming to update recall responses to the relevant influenza strain while retaining the ability to generate more broadly reactive antibodies without heavily imprinting the response against one strain (56).

αMHCII-CA07 induced a higher number of HA-specific GC B cells than Pandemrix. Interestingly, even αMHCII-CA07 without AS03 raised responses comparable to those from Pandemrix (Fig. 6E). That said, Pandemrix induced more GC B cells in total than any of the other vaccines, likely due to the presence of many viral antigens in this split virion vaccine (Fig. S4B). Both Flublok and αMHCII-CA07+AS03 raised higher numbers of HA-specific GC B cells (30) (Fig. 6E), highlighting that subunit vaccines have a benefit in activating immune responses specifically against the desired antigen. In support of the latter point, Flublok could mediate equal protection from disease compared to the adjuvanted Pandemrix, and even the reduced dose of αMHCII-CA07+AS03 awarded protection, albeit with some initial weight loss (Fig. 6C). Another factor may be the interaction between vaccine antigens and specific immune cells. It is known that AS03 leads to preferential loading of monocytes over dendritic cells in draining lymph nodes (57), while we have previously seen that MHCII-targeted antigen (in DNA vaccine format) binds both macrophages and dendritic cells at roughly equal levels (13, 30).

We have here demonstrated that APC-targeted subunit vaccines can mimic the natural HA conformation and produce trimeric HA even in the absence of a trimerization domain. Importantly, the vaccines have demonstrated immunogenicity and functional efficacy against viral challenges in the forms of both DNA and protein. While the present data are from mice, the result warrants progression to larger animals as a first step toward clinical progression. The vaccines may be of use to reduce the seasonal burdens of influenza disease in the population, but our experiments also demonstrated a potential against emerging influenza subtypes with a pandemic potential.

## MATERIALS AND METHODS

### Cloning of vaccine constructs.

A DNA sequence encoding an MHCII-specific scFv (originating from clone 14-4-4S MAb [22, 27), a short linker (GESYAEAAAKEAAAK), and HA (amino acids 18 to 541, codon optimized for mammalian protein production) from influenza A/PR/8/1934 (H1N1) (PR8, denoted H1) (13), followed by a linker (LNDIFEAQKIEWHERLVPRGS) and a foldon trimerization domain (PGSGYIPEAPRDGQAYVRKDGEWVLLSTFLG, denoted in vaccine names as H1F) was ordered for synthesis by GenScript (New Jersey, USA) (Fig. 1A). The construct was cloned into pLNOH2 (58). Vaccines encoding mammalian codon-optimized HA antigens from influenza A/Vietnam/1194/2004(H5N1) (VN04), A/Hong Kong/1073/99(H9N2) (HK99), and A/California/07/2009(H1N1) were constructed by replacing the PR8 HA by subcloning on antigen-flanking SfiI-sites. Further, vaccines encoding the murine macrophage inflammatory protein 1 alpha (MIP1α), or a nontargeting scFv against the hapten 4-hydroxy-3-iodo-5-nitrophenylacetic acid (NIP) (originating from clone B1-8 MAb [27]) as a nontargeted control vaccine, were prepared by replacing the MHCII-specific scFv with subcloning on the BsmI/BsiWI restriction sites.

### Recombinant protein vaccine production.

Vaccine plasmids were amplified in TOP10 Escherichia coli bacteria and purified using either the Wizard Plus SV miniprep DNA purification system (catalog [cat.] no. A1460, Promega, WI, USA) for small quantities or the Qiagen plasmid mega-kit (cat. no. 12181, Qiagen, Germany) for larger quantities. For recombinant protein production, HEK293E cells were transfected with 0.25 μg plasmid per cm^2^ cell tissue surface at 70% confluence with 40 μg polyethyleneimine (PEI) per μg plasmid DNA in 40 μL Opti-MEM (cat. no. 51985-026, Thermo Fisher, Massachusetts, USA) per μg plasmid DNA. Serum free Freestyle medium (cat. no. 12338018, Thermo Fisher) was used for all transfections. Cell culture medium was harvested 3 to 5 days posttransfection for small-scale production and every 5 days for large-scale transfections. For affinity purification of vaccine protein, filtered cell culture medium was applied to a protein A column loaded with either PR8-specific (clone H36-4-52, kind gift from Siegfried Weiss, Medizinische Hochschule, Hannover, Germany) or CA07-specific (clone 29E3, kind gift from Thomas Moran, Icahn School of Medicine at Mount Sinai, New York, NY, USA) monoclonal antibodies, followed by elution using 0.1 M Tris-glycine, pH 2.7. Buffer exchange was performed by dialysis with phosphate-buffered saline (PBS) using Spectra/Por dialysis membrane MWCO 12-14,000 (cat. no. 132 697, Repligen, Massachusetts, USA).

### Structure characterization: MHCII (I-E^d^) binding analysis.

MHCII-transfected L-cell fibroblasts expressing E_β_^d^E_α_^k^ (CA36.2.1) or D^d^ (CA25.8.2) (kind gift from Bernard Malissen, Centre d’Immunophénomique, Aix Marseille Université, Marseille, France) were stained with purified vaccine protein (10 μg/mL) for 30 min at 4°C, followed by incubation with biotinylated anti-HA IgG (1 μg/mL; clone: H36-4-52) and incubation with streptavidin-phycoerythrin (PE) (1 μg/mL; cat. no. S866, Thermo Fisher). Flow analysis was performed on an Attune NxT instrument (Thermo Fisher), and data analysis was performed in FlowJo (FlowJo LLC, BD, New Jersey, USA).

### Structure characterization: chemotaxis.

The chemotactic integrity of MIP1α-H1/H1F was assessed by quantifying Esb/MP cell migration across a 5-μm-pore polycarbonate membrane (cat. no. 3421, Corning, Inc., New York, USA) in response to the titrated presence of vaccine proteins. Results from duplicate samples (mean) after background subtraction (mean cell numbers of spontaneous cell migration, i.e., in the presence of medium alone) are presented.

### Structure characterization: enzyme-linked immunosorbent assay (ELISA).

High-binding 96-well microtiter plates (Costar 3590, Corning, NY, USA) were coated with 50 μL of NIP-bovine serum albumin (BSA) (2 μg/mL) overnight at 4°C and blocked with 1% BSA-PBS for 60 min at room temperature. Next, 50 μL of supernatant from HEK293E cells transiently transfected with αNIP-H1(-F) or mock was added and incubated for 2 h at room temperature. This step was repeated for saturation. Next, titrated dilutions of the following antibody panel (kind gift from Davide Angeletti and Jonathan W. Yewdell, National Institute of Allergy and Infectious Diseases, National Institutes of Health, Bethesda, MD, USA) (36) were incubated in triplicates at 4°C overnight: Y8-3B3, Y8-2C6, H2-4B3, H28-E23, H36-1-1, H35-D1, H17-L2, H37-80, H18-S121, H2-4B1, H18-S413, H36-11, H17-L7, H9-A15, L2-10C1, D5, E7, 16GB, and H36-4-52. Then, plates were incubated for 2 h with biotinylated MAb specific for mouse κ-light chain (clone: 187, produced in-house) at room temperature, followed by streptavidin-alkaline phosphatase conjugate (ALP) (1:30,00; cat. no. 7105-04, SouthernBiotech, Alabama, USA) for 30 min. Detection was done following 15 min of incubation with phosphatase substrate (cat. no. P4744-10G, Merck, New Jersey, USA) at 405 nm. Antibody binding was considered positive if the area under the curve (AUC) of the dilution was higher than the AUC + 5 × standard error of the mean (SEM) of a mock transfection for that antibody. H1 PR8 vaccine secretion was measured using an influenza A H1N1 (A/Puerto Rico/8/1934) hemagglutinin/HA ELISA pair set (cat. no. SEK11684, Sino Biological, China), according to the manufacturer’s instructions.

### Structure characterization: Western blotting.

Purified vaccine proteins were either kept native or mixed with 0.1 M dithiothreitol (DTT) and heated to 95°C for 5 min. Vaccine proteins (0.1 μg per lane) were loaded on 4 to 12% NuPAGE bis-tris gels (cat. no. NP0326BOX, Thermo Fisher, Massachusetts, USA) and separated with Bolt MOPS (morpholinepropanesulfonic acid) SDS running buffer (cat. no. B0001, Thermo Fisher). Proteins were then transferred to iBlot 2 polyvinylidene difluoride (PVDF) transfer stacks (cat. no. IB24002, Thermo Fisher), blocked in 2% skim milk in PBS with Tween 20 (PBST) at room temperature for 60 min, and incubated with biotinylated anti-HA MAb (1:3,000; clone: H36-4-52) at 4°C for 18 h. Next, the blot was incubated with streptavidin-horseradish peroxidase (HRP; 1:10,000; cat. no. 7105-05, SouthernBiotech) for 30 min at room temperature. Detection was done with WestPico chemiluminescence substrate (cat. no. 34578, Thermo Fisher). Separately, recombinant PR8 protein was deglycosylated with peptide-*N*-glycosidase F (PNGase F) according to the manufacturer’s instructions (cat no. P0704S, New England Biolabs, Massachusetts, USA) to determine the size contribution of glycan moieties on HA.

### Structure characterization: analytical size exclusion chromatography.

Purified recombinant vaccine proteins (10 ng) or 10 ng of a commercially available HA (cat. no. 11684-V08H, Sino Biologicals) was loaded onto a Superdex 200 Increase 3.2/300 HPLC chromatography system (cat. no. 28990946, GE Healthcare, Sweden) in PBS. Molecular weights (MW) were compared against a ladder of proteins with known size (MWGF-1000-1KT, Merck, New Jersey, USA).

### Animals, *in vivo* immunization, and viral challenge.

All animals used in this study were female BALB/c mice (Janvier Labs, France) housed in a minimal disease unit at Oslo University Hospital. All experiments were approved by the Norwegian Animal Research Authority.

Mice were anaesthetized by an intraperitoneal (i.p.) injection of ZRF (zolazepam [3.3 mg/mL], tiletamine [3.3 mg/mL], xylazine [0.45 mg/mL], fentanyl [2.6 μg/mL]) at 10 μL/g body weight. Immunizations were performed by first shaving the hind legs of anaesthetized mice, followed by an intramuscular (i.m.) injection of 50 μL DNA (0.5 mg/mL) solution into each quadriceps, immediately followed by five-pulse electroporation of the injection site with an AgilePulse system (Harvard Apparatus BTX, Holliston, MA). Protein vaccination was performed under anesthesia by i.m. injection of 50 μL vaccine solution in each quadriceps. For commercial vaccines, 2 × 50 μL of Flublok (Sanofi Pasteur, France) or a 1:1 mixture of Pandemrix:AS03 (GSK, Belgium) was delivered i.m. Viral influenza challenges were done by infecting anaesthetized mice intranasally (i.n.) with a virus dose of 5× the 50% lethal dose (LD_50_) in 10 μL per nostril. The LD_50_ dose was established by the Reed and Muench method and titrations of virus in mice. The viral strains used in this study were A/Puerto Rico/8/1934(H1N1), A/California/07/2009(H1N1), and RG14 [reassorted PR8 virus with H5 from A/Viet Nam/04/2005(H5N1)].

### Serum Ab titer by ELISA.

Blood samples were taken by puncture of the saphenous vein. Serum was obtained by centrifugation at 17,000 × *g* for 10 min, and then the supernatant was transferred to a new microcentrifuge tube and centrifuged again at 17,000 × *g* for 5 min. Sera were stored at −20°C.

For ELISA, 96-well microtiter plates (Costar 3590, Corning, NY, USA) were coated with one of the following recombinant influenza HA proteins: A/Puerto Rico/8/1934(H1N1) (cat. no. 11684-V08H, Sino Biological), A/Viet Nam/1194/2004(H5N1) (cat. no. 11062-V08H1, Sino Biological), or A/Hong Kong/1073/99(H9N2) (cat. no. 11229-V08H, Sino Biological) (0.5 μg/mL). Plates were blocked by 1% BSA in PBS at room temperature for 1 h. Serially diluted sera from individual mice were then incubated at 4°C for 16 to 18 h. Murine IgG was detected by incubation for 1 h with either horseradish peroxidase (HRP)-conjugated anti-mouse IgG (Fc specific) (1:5,000; cat. no. A2554, Merck, New Jersey, USA), biotinylated anti-mouse IgG1[a] (1:500; cat. no. 553500, BD Biosciences, New Jersey, USA), or biotinylated anti-mouse IgG2a[a] (1:500; cat. no. 553504, BD Biosciences). For biotinylated antibodies, a secondary incubation with HRP-conjugated streptavidin (1:5,000; cat.no. 7105-05, SouthernBiotech, Alabama, USA) at room temperature for 30 min was performed. The plates were developed by addition of 3,3′,5,5′-tetramethylbenzidine (TMB) solution (cat. no. CL07-1000ML, Merck), and the reaction was stopped after 10 min by addition of 1 M H_2_SO_4_. Absorbance at 450 nm was read using a Wallac EnVision 2104 Multilabel Reader (PerkinElmer, Massachusetts, USA).

### Enzyme-linked immunosorbent spot (ELISpot).

Spleens were harvested 14 days postimmunization. Single cell suspensions were prepared by using a gentleMACS dissociator (Miltenyi Biotec, Germany) followed by incubation in ACT (150 mM NH_4_Cl, 170 mM TRIS-Base, pH 7.2) for 10 min on ice before filtration through a 70-μm nylon cell strainer (cat. no. 732-2758, VWR, Pennsylvania, USA). Splenocytes were washed twice with PBS before counting and resuspension in RPMI 1640 plus 10% FBS (cat. no. 61870036, Thermo Fisher). Cells from each mouse were then mixed with different stimuli in triplicates and assayed in accordance with the kit protocol (cat. no. 3321-4APT, ELISpot Plus, Mabtech, Sweden) for 18 h at 37°C in a humidified atmosphere with 5% CO_2_. The stimuli were recombinant (rec). HA from influenza PR8 or Cal07 (10 μg/mL; cat. no. 11684-V08H/11055-V08H, Sino Biological), ovalbumin (10 μg/mL; cat. no. vac-pova-100, InvivoGen, California, USA), IYSTVASSL peptide (7 μg/mL; ThinkPeptides, United Kingdom), PR8 HA peptide pool (15-mers with 11-amino acid [aa] overlap) (7 μg/mL; PepMix influenza A [HA/Puerto Rico/8/1934 H1N1]; cat. no. PM-INFA-HAPR, JPT Peptide Technologies, Germany), CA07 HA peptide pool (15-mers with 11-aa overlap) (7 μg/mL; PepMix influenza A [HA/California (H1N1)]; cat. no. PM-INFA-HACal, JPT Peptide Technologies), ConA (1 to 2.5 μg/mL; cat. no. inh-cona, InvivoGen), and RPMI 1640 (cat. no. 61870036, Thermo Fisher, Massachusetts, USA). The spots were counted in an ImmunoSpot device (C.T.L Cellular Technologies Limited, Ohio, USA).

### Flow cytometry.

Single cell suspensions were obtained from freshly harvested inguinal lymph nodes by passage through a 70-μm nylon strainer (cat. no. 732-2758, VWR). The cells were washed with PBS, and 1 × 10^6^ cells were blocked on ice with 50% rat serum in 0.5% BSA/PBS for 30 min. The cells were then stained with a cocktail of anti-T-cell receptor β (TCRβ)-Alexa Fluor 488 (1:200; cat. no. 109201, BioLegend, California, USA), anti-CD45R/B220-PerCP/Cy5.5 (1:200; cat. no. 65-0452-U100, Tonbo Biosciences, California, USA), anti-CD19-APC/Cy7 (1:200; cat. no. 125530, BioLegend), anti-CD38-APC (1:200; cat. no. 102711, BioLegend), and anti-HU/MU GL7-Pacific Blue (1:200; cat. no.141614, BioLegend). In addition, purified recombinant HA CA07_Y96F_ with an AviTag (GGGLNDIFEAQKIEWHE) was biotinylated (cat. no. BIRA500, Avidity, Colorado, USA) and then coupled at a molar ratio of 5:1 with streptavidin-PE (cat. no. 405204, BioLegend) at 4°C for 16 h or 15:1 with Klickmer-PE (cat. no. DX01K-PE, Immudex, Denmark) at room temperature for 30 min. The resulting HA complex was added to the staining cocktail at 1:100 for HA-SA-PE or at 32 nM HA-Klickmer-PE per sample. Flow analysis was performed on an Attune NxT instrument (Thermo Fisher), and data analysis was performed in FlowJo (FlowJo LLC, BD, New Jersey, USA). Cells were gated as lymphocytes, singlets, TCR_β_^neg^ (non-T cells), B220^pos^ CD19^pos^ (B cells), CD38^neg^ GL7^int-hi^ (GC B cells), and HA^pos^.

### Statistical analysis.

Serum antibody levels, protein secretion, and ELISpot counts were analyzed by using a pairwise two-sided Mann-Whitney test at each time point. Survival rates were analyzed by log-rank (Mantel-Cox) and Gehan-Breslow-Wilcoxon tests. All statistical analyses were performed in GraphPad Prism (California, USA).

## References

[1] Yamada S, Suzuki Y, Suzuki T, Le MQ, Nidom CA, Sakai-Tagawa Y, Muramoto Y, Ito M, Kiso M, Horimoto T, Shinya K, Sawada T, Kiso M, Usui T, Murata T, Lin Y, Hay A, Haire LF, Stevens DJ, Russell RJ, Gamblin SJ, Skehel JJ, Kawaoka Y. 2006. Haemagglutinin mutations responsible for the binding of H5N1 influenza A viruses to human-type receptors. Nature 444:378–382. 10.1038/nature05264. 17108965

[2] Caffrey M, Lavie A. 2021. pH-dependent mechanisms of influenza infection mediated by hemagglutinin. Front Mol Biosci 8:777095. 10.3389/fmolb.2021.777095. 34977156 PMC8718792

[3] Gerhard W, Yewdell J, Frankel ME, Webster R. 1981. Antigenic structure of influenza virus haemagglutinin defined by hybridoma antibodies. Nature 290:713–717. 10.1038/290713a0. 6163993

[4] Padilla-Quirarte HO, Lopez-Guerrero DV, Gutierrez-Xicotencatl L, Esquivel-Guadarrama F. 2019. Protective antibodies against influenza proteins. Front Immunol 10:1677. 10.3389/fimmu.2019.01677. 31379866 PMC6657620

[5] Xu R, Ekiert DC, Krause JC, Hai R, Crowe JE, Jr, Wilson IA. 2010. Structural basis of preexisting immunity to the 2009 H1N1 pandemic influenza virus. Science 328:357–360. 10.1126/science.1186430. 20339031 PMC2897825

[6] Tsibane T, Ekiert DC, Krause JC, Martinez O, Crowe JE, Jr, Wilson IA, Basler CF. 2012. Influenza human monoclonal antibody 1F1 interacts with three major antigenic sites and residues mediating human receptor specificity in H1N1 viruses. PLoS Pathog 8:e1003067. 10.1371/journal.ppat.1003067. 23236279 PMC3516549

[7] Wall DJ, Patel MM, Chung JR, Lee B, Dawood FS. 2021. Antibody response and protection after receipt of inactivated influenza vaccine: a systematic review. Pediatrics 147:e2020019901. 10.1542/peds.2020-019901. 34039716

[8] Moa AM, Chughtai AA, Muscatello DJ, Turner RM, MacIntyre CR. 2016. Immunogenicity and safety of inactivated quadrivalent influenza vaccine in adults: a systematic review and meta-analysis of randomised controlled trials. Vaccine 34:4092–4102. 10.1016/j.vaccine.2016.06.064. 27381642

[9] Osterholm MT, Kelley NS, Sommer A, Belongia EA. 2012. Efficacy and effectiveness of influenza vaccines: a systematic review and meta-analysis. Lancet Infect Dis 12:36–44. 10.1016/S1473-3099(11)70295-X. 22032844

[10] Murchu EO, Comber L, Jordan K, Hawkshaw S, Marshall L, O’Neill M, Ryan M, Teljeur C, Carnahan A, Pérez JJ, Robertson AH, Johansen K, de Jonge J, Krause T, Nicolay N, Nohynek H, Pavlopoulou I, Pebody R, Penttinen P, Soler-Soneira M, Wichmann O, Harrington P. 2022. Systematic review of the efficacy, effectiveness and safety of MF59 adjuvanted seasonal influenza vaccines for the prevention of laboratory-confirmed influenza in individuals ≥18 years of age. Rev Med Virol 10.1002/rmv.2329. 35142401

[11] Comber L, Murchu EO, Jordan K, Hawkshaw S, Marshall L, O’Neill M, Teljeur C, Ryan M, Carnahan A, Pérez Martín JJ, Robertson AH, Johansen K, Jonge J, Krause T, Nicolay N, Nohynek H, Pavlopoulou I, Pebody R, Penttinen P, Soler-Soneira M, Wichmann O, Harrington P. 2022. Systematic review of the efficacy, effectiveness and safety of high-dose seasonal influenza vaccines for the prevention of laboratory-confirmed influenza in individuals ≥18 years of age. Rev Med Virol 10.1002/rmv.2330. 35119149

[12] Zlatkovic J, Stiasny K, Heinz FX. 2011. Immunodominance and functional activities of antibody responses to inactivated West Nile virus and recombinant subunit vaccines in mice. J Virol 85:1994–2003. 10.1128/JVI.01886-10. 21147919 PMC3067796

[13] Grodeland G, Mjaaland S, Roux KH, Fredriksen AB, Bogen B. 2013. DNA vaccine that targets hemagglutinin to MHC class II molecules rapidly induces antibody-mediated protection against influenza. J Immunol 191:3221–3231. 10.4049/jimmunol.1300504. 23956431 PMC3767367

[14] Xuan C, Shi Y, Qi J, Zhang W, Xiao H, Gao GF. 2011. Structural vaccinology: structure-based design of influenza A virus hemagglutinin subtype-specific subunit vaccines. Protein Cell 2:997–1005. 10.1007/s13238-011-1134-y. 22231357 PMC4875251

[15] Eckshtain-Levi M, Batty CJ, Lifshits LM, McCammitt B, Moore KM, Amouzougan EA, Stiepel RT, Duggan E, Ross TM, Bachelder EM, Ainslie KM. 2022. Metal-organic coordination polymer for delivery of a subunit broadly acting influenza vaccine. ACS Appl Mater Interfaces 14:28548–28558. 10.1021/acsami.2c04671. 35704854 PMC9495290

[16] Carter DM, Darby CA, Lefoley BC, Crevar CJ, Alefantis T, Oomen R, Anderson SF, Strugnell T, Cortés-Garcia G, Vogel TU, Parrington M, Kleanthous H, Ross TM. 2016. Design and characterization of a computationally optimized broadly reactive hemagglutinin vaccine for H1N1 influenza viruses. J Virol 90:4720–4734. 10.1128/JVI.03152-15. 26912624 PMC4836330

[17] Ross TM, Xu Y, Bright RA, Robinson HL. 2000. C3d enhancement of antibodies to hemagglutinin accelerates protection against influenza virus challenge. Nat Immunol 1:127–131. 10.1038/77802. 11248804 PMC1635154

[18] Watanabe A, McCarthy KR, Kuraoka M, Schmidt AG, Adachi Y, Onodera T, Tonouchi K, Caradonna TM, Bajic G, Song S, McGee CE, Sempowski GD, Feng F, Urick P, Kepler TB, Takahashi Y, Harrison SC, Kelsoe G. 2019. Antibodies to a conserved influenza head interface epitope protect by an IgG subtype-dependent mechanism. Cell 177:1124–1135.e16. 10.1016/j.cell.2019.03.048. 31100267 PMC6825805

[19] Krammer F, Margine I, Tan GS, Pica N, Krause JC, Palese P. 2012. A carboxy-terminal trimerization domain stabilizes conformational epitopes on the stalk domain of soluble recombinant hemagglutinin substrates. PLoS One 7:e43603. 10.1371/journal.pone.0043603. 22928001 PMC3426533

[20] Weldon WC, Wang B-Z, Martin MP, Koutsonanos DG, Skountzou I, Compans RW. 2010. Enhanced immunogenicity of stabilized trimeric soluble influenza hemagglutinin. PLoS One 5:e12466. 10.1371/journal.pone.0012466. 20824188 PMC2931692

[21] Magadán JG, Khurana S, Das SR, Frank GM, Stevens J, Golding H, Bennink JR, Yewdell JW. 2013. Influenza A virus hemagglutinin trimerization completes monomer folding and antigenicity. J Virol 87:9742–9753. 10.1128/JVI.00471-13. 23824811 PMC3754138

[22] Carayanniotis G, Barber BH. 1987. Adjuvant-free IgG responses induced with antigen coupled to antibodies against class II MHC. Nature 327:59–61. 10.1038/327059a0. 3472080

[23] Ho J, MacDonald KS, Barber BH. 2002. Construction of recombinant targeting immunogens incorporating an HIV-1 neutralizing epitope into sites of differing conformational constraint. Vaccine 20:1169–1180. 10.1016/S0264-410X(01)00441-8. 11803079

[24] Biragyn A, Tani K, Grimm MC, Weeks S, Kwak LW. 1999. Genetic fusion of chemokines to a self tumor antigen induces protective, T-cell dependent antitumor immunity. Nat Biotechnol 17:253–258. 10.1038/6995. 10096292

[25] Biragyn A, Ruffini PA, Coscia M, Harvey LK, Neelapu SS, Baskar S, Wang J-M, Kwak LW. 2004. Chemokine receptor-mediated delivery directs self-tumor antigen efficiently into the class II processing pathway in vitro and induces protective immunity in vivo. Blood 104:1961–1969. 10.1182/blood-2004-02-0637. 15191951

[26] Schiavo R, Baatar D, Olkhanud P, Indig FE, Restifo N, Taub D, Biragyn A. 2006. Chemokine receptor targeting efficiently directs antigens to MHC class I pathways and elicits antigen-specific CD8+ T-cell responses. Blood 107:4597–4605. 10.1182/blood-2005-08-3207. 16514063 PMC1895803

[27] Fredriksen AB, Sandlie I, Bogen B. 2006. DNA vaccines increase immunogenicity of idiotypic tumor antigen by targeting novel fusion proteins to antigen-presenting cells. Mol Ther 13:776–785. 10.1016/j.ymthe.2005.10.019. 16414309

[28] Grødeland G, Mjaaland S, Tunheim G, Fredriksen AB, Bogen B. 2013. The specificity of targeted vaccines for APC surface molecules influences the immune response phenotype. PLoS One 8:e80008. 10.1371/journal.pone.0080008. 24244595 PMC3823800

[29] Grødeland G, Fossum E, Bogen B. 2015. Polarizing T and B cell responses by APC-targeted subunit vaccines. Front Immunol 6:367. 10.3389/fimmu.2015.00367. 26257735 PMC4507452

[30] Andersen TK, Huszthy PC, Gopalakrishnan RP, Jacobsen JT, Fauskanger M, Tveita AA, Grødeland G, Bogen B. 2019. Enhanced germinal center reaction by targeting vaccine antigen to major histocompatibility complex class II molecules. NPJ Vaccines 4:9. 10.1038/s41541-019-0101-0. 30775000 PMC6370881

[31] Grodeland G, Fredriksen AB, Løset GÅ, Vikse E, Fugger L, Bogen B. 2016. Antigen targeting to human HLA class II molecules increases efficacy of DNA vaccination. J Immunol 197:3575–3585. 10.4049/jimmunol.1600893. 27671110 PMC5073356

[32] Hinke DM, Andersen TK, Gopalakrishnan RP, Skullerud LM, Werninghaus IC, Grødeland G, Fossum E, Braathen R, Bogen B. 2022. Antigen bivalency of antigen-presenting cell-targeted vaccines increases B cell responses. Cell Rep 39:110901. 10.1016/j.celrep.2022.110901. 35649357

[33] Braathen R, Spång HCL, Lindeberg MM, Fossum E, Grødeland G, Fredriksen AB, Bogen B. 2018. The magnitude and IgG subclass of antibodies elicited by targeted DNA vaccines are influenced by specificity for APC surface molecules. Immunohorizons 2:38–53. 10.4049/immunohorizons.1700038. 31022690

[34] Tesini BL, Kanagaiah P, Wang J, Hahn M, Halliley JL, Chaves FA, Nguyen PQT, Nogales A, DeDiego ML, Anderson CS, Ellebedy AH, Strohmeier S, Krammer F, Yang H, Bandyopadhyay S, Ahmed R, Treanor JJ, Martinez-Sobrido L, Golding H, Khurana S, Zand MS, Topham DJ, Sangster MY. 2019. Broad hemagglutinin-specific memory B cell expansion by seasonal influenza virus infection reflects early-life imprinting and adaptation to the infecting virus. J Virol 93:e00169-19. 10.1128/JVI.00169-19. 30728266 PMC6450111

[35] Fredriksen AB, Bogen B. 2007. Chemokine-idiotype fusion DNA vaccines are potentiated by bivalency and xenogeneic sequences. Blood 110:1797–1805. 10.1182/blood-2006-06-032938. 17540847

[36] Angeletti D, Gibbs JS, Angel M, Kosik I, Hickman HD, Frank GM, Das SR, Wheatley AK, Prabhakaran M, Leggat DJ, McDermott AB, Yewdell JW. 2017. Defining B cell immunodominance to viruses. Nat Immunol 18:456–463. 10.1038/ni.3680. 28192417 PMC5360521

[37] Mallapaty S. 2021. India’s DNA COVID vaccine is a world first – more are coming. Nature 597:161–162. 10.1038/d41586-021-02385-x. 34475553

[38] Huber VC, McCullers JA. 2008. FluBlok, a recombinant influenza vaccine. Curr Opin Mol Ther 10:75–85. 18228185

[39] Walker WT, Faust SN. 2010. Monovalent inactivated split-virion AS03-adjuvanted pandemic influenza A (H1N1) vaccine. Expert Rev Vaccines 9:1385–1398. 10.1586/erv.10.141. 21105775

[40] Caton AJ, Brownlee GG, Yewdell JW, Gerhard W. 1982. The antigenic structure of the influenza virus A/PR/8/34 hemagglutinin (H1 subtype). Cell 31:417–427. 10.1016/0092-8674(82)90135-0. 6186384

[41] Dintzis HM, Dintzis RZ, Vogelstein B. 1976. Molecular determinants of immunogenicity: the immunon model of immune response. Proc Natl Acad Sci USA 73:3671–3675. 10.1073/pnas.73.10.3671. 62364 PMC431180

[42] Pone EJ, Hernandez-Davies JE, Jan S, Silzel E, Felgner PL, Davies DH. 2022. Multimericity amplifies the synergy of BCR and TLR4 for B cell activation and antibody class switching. Front Immunol 13:882502. 10.3389/fimmu.2022.882502. 35663959 PMC9161726

[43] Grødeland G, Baranowska-Hustad M, Abadejos J, Blane TR, Teijaro J, Nemazee D, Bogen B. 2020. Induction of cross-reactive and protective antibody responses after DNA vaccination with MHCII-targeted stem domain from influenza hemagglutinin. Front Immunol 11:431. 10.3389/fimmu.2020.00431. 32269566 PMC7112135

[44] Seephetdee C, Buasri N, Bhukhai K, Srisanga K, Manopwisedjaroen S, Lertjintanakit S, Phueakphud N, Pakiranay C, Kangwanrangsan N, Srichatrapimuk S, Kirdlarp S, Sungkanuparph S, Chutipongtanate S, Thitithanyanont A, Hongeng S, Wongtrakoongate P. 2021. Mice immunized with the vaccine candidate HexaPro spike produce neutralizing antibodies against SARS-CoV-2. Vaccines 9:498. 10.3390/vaccines9050498. 34066016 PMC8151071

[45] World Health Organization. 2006. Update: WHO-confirmed human cases of avian influenza A(H5N1) infection. https://www.who.int/publications/i/item/who-wer-8206-41-47. Accessed 16 June 2022.

[46] World Health Organization. 2006. Epidemiology of WHO-confirmed human cases of avian influenza A(H5N1) infection. https://www.who.int/publications-detail-redirect/who-wer-8126-249-257. Accessed 16 June 2022.

[47] Jackson LA, Anderson EJ, Rouphael NG, Roberts PC, Makhene M, Coler RN, McCullough MP, Chappell JD, Denison MR, Stevens LJ, Pruijssers AJ, McDermott A, Flach B, Doria-Rose NA, Corbett KS, Morabito KM, O’Dell S, Schmidt SD, Swanson PA, Padilla M, Mascola JR, Neuzil KM, Bennett H, Sun W, Peters E, Makowski M, Albert J, Cross K, Buchanan W, Pikaart-Tautges R, Ledgerwood JE, Graham BS, Beigel JH, mRNA-1273 Study Group. 2020. An mRNA vaccine against SARS-CoV-2: preliminary report. N Engl J Med 383:1920–1931. 10.1056/NEJMoa2022483. 32663912 PMC7377258

[48] Polack FP, Thomas SJ, Kitchin N, Absalon J, Gurtman A, Lockhart S, Perez JL, Marc GP, Moreira ED, Zerbini C, Bailey R, Swanson KA, Roychoudhury S, Koury K, Li P, Kalina WV, Cooper D, Frenck RW, Hammitt LL, Türeci Ö, Nell H, Schaefer A, Ünal S, Tresnan DB, Mather S, Dormitzer PR, Şahin U, Jansen KU, Gruber WC, C4591001 Clinical Trial Group. 2020. Safety and efficacy of the BNT162b2 mRNA Covid-19 vaccine. N Engl J Med 383:2603–2615. 10.1056/NEJMoa2034577. 33301246 PMC7745181

[49] Momin T, Kansagra K, Patel H, Sharma S, Sharma B, Patel J, Mittal R, Sanmukhani J, Maithal K, Dey A, Chandra H, Rajanathan CT, Pericherla HP, Kumar P, Narkhede A, Parmar D. 2021. Safety and immunogenicity of a DNA SARS-CoV-2 vaccine (ZyCoV-D): results of an open-label, non-randomized phase I part of phase I/II clinical study by intradermal route in healthy subjects in India. EClinicalMedicine 38:101020. 10.1016/j.eclinm.2021.101020. 34308319 PMC8285262

[50] Food and Drug Administration. 2021. FDA approves first COVID-19 vaccine. https://www.fda.gov/news-events/press-announcements/fda-approves-first-covid-19-vaccine. Accessed 14 March 2022.

[51] European Medicines Agency. 2020. EMA recommends first COVID-19 vaccine for authorization in the EU. https://www.ema.europa.eu/en/news/ema-recommends-first-covid-19-vaccine-authorisation-eu. Accessed 14 March 2022.

[52] Cai C, Peng Y, Shen E, Huang Q, Chen Y, Liu P, Guo C, Feng Z, Gao L, Zhang X, Gao Y, Liu Y, Han Y, Zeng S, Shen H. 2021. A comprehensive analysis of the efficacy and safety of COVID-19 vaccines. Mol Ther J Am Soc Gene Ther 29:2794–2805. 10.1016/j.ymthe.2021.08.001. PMC8342868 34365034

[53] Aldén M, Falla FO, Yang D, Barghouth M, Luan C, Rasmussen M, De Marinis Y. 2022. Intracellular reverse transcription of Pfizer BioNTech COVID-19 mRNA vaccine BNT162b2 in vitro in human liver cell line. Curr Issues Mol Biol 44:1115–1126. 10.3390/cimb44030073. 35723296 PMC8946961

[54] Wang Z, Troilo PJ, Wang X, Griffiths TG, Pacchione SJ, Barnum AB, Harper LB, Pauley CJ, Niu Z, Denisova L, Follmer TT, Rizzuto G, Ciliberto G, Fattori E, Monica NL, Manam S, Ledwith BJ. 2004. Detection of integration of plasmid DNA into host genomic DNA following intramuscular injection and electroporation. Gene Ther 11:711–721. 10.1038/sj.gt.3302213. 14724672

[55] Hallberg P, Smedje H, Eriksson N, Kohnke H, Daniilidou M, Öhman I, Yue Q-Y, Cavalli M, Wadelius C, Magnusson PKE, Landtblom A-M, Wadelius M, Swedegene. 2019. Pandemrix-induced narcolepsy is associated with genes related to immunity and neuronal survival. EBioMedicine 40:595–604. 10.1016/j.ebiom.2019.01.041. 30711515 PMC6413474

[56] Röltgen K, Nielsen SCA, Silva O, Younes SF, Zaslavsky M, Costales C, Yang F, Wirz OF, Solis D, Hoh RA, Wang A, Arunachalam PS, Colburg D, Zhao S, Haraguchi E, Lee AS, Shah MM, Manohar M, Chang I, Gao F, Mallajosyula V, Li C, Liu J, Shoura MJ, Sindher SB, Parsons E, Dashdorj NJ, Dashdorj ND, Monroe R, Serrano GE, Beach TG, Chinthrajah RS, Charville GW, Wilbur JL, Wohlstadter JN, Davis MM, Pulendran B, Troxell ML, Sigal GB, Natkunam Y, Pinsky BA, Nadeau KC, Boyd SD. 2022. Immune imprinting, breadth of variant recognition, and germinal center response in human SARS-CoV-2 infection and vaccination. Cell 185:1025–1040.e14. 10.1016/j.cell.2022.01.018. 35148837 PMC8786601

[57] Morel S, Didierlaurent A, Bourguignon P, Delhaye S, Baras B, Jacob V, Planty C, Elouahabi A, Harvengt P, Carlsen H, Kielland A, Chomez P, Garçon N, Van Mechelen M. 2011. Adjuvant system AS03 containing α-tocopherol modulates innate immune response and leads to improved adaptive immunity. Vaccine 29:2461–2473. 10.1016/j.vaccine.2011.01.011. 21256188

[58] Norderhaug L, Olafsen T, Michaelsen TE, Sandlie I. 1997. Versatile vectors for transient and stable expression of recombinant antibody molecules in mammalian cells. J Immunol Methods 204:77–87. 10.1016/S0022-1759(97)00034-3. 9202712

