# Supplementary material for: Trimeric, APC-Targeted Subunit Vaccines Protect Mice against Seasonal and Pandemic Influenza
Source: J Virol. 2023 Jan 31;97(2):e01694-22. doi: 10.1128/jvi.01694-22 (PMC9972960; doi:10.1128/jvi.01694-22)
Supplement: Supplemental file 1 — Table S1 and Fig. S1 to S4. Download jvi.01694-22-s0001.pdf, PDF file, 0.6 MB [file jvi.01694-22-s0001.pdf]

Supplementary Material

Supplementary Table I: Overview of the constructed vaccines

Vaccine number	Name	Targeting domain	Antigen	Gene Accession No	C-term Foldon
1	αMHCII-H1-F	Anti-MHCII	H1(A/Puerto Rico/8/1934)	V01088	yes
2	αMHCII-H1	Anti-MHCII	H1(A/Puerto Rico/8/1934)	V01088	no
3	MIP1α-H1-F	MIP1α (CCL3)	H1(A/Puerto Rico/8/1934)	V01088	yes
4	MIP1α -H1	MIP1α (CCL3)	H1(A/Puerto Rico/8/1934)	V01088	no
5	αNIP-H1-F	αNIP	H1(A/Puerto Rico/8/1934)	V01088	yes
6	αNIP-H1	αNIP	H1(A/Puerto Rico/8/1934)	V01088	no
7	αMHCII-CA07	Anti-MHCII	H1(A/California/07/2009)	CY266191	no
8	αMHCII-H5	Anti-MHCII	H5(A/Vietnam/1194/2004)	EF541402	no
9	αMHCII-H9	Anti-MHCII	H9(A/Hong Kong/1073/99(H9N2)	AJ404626	no

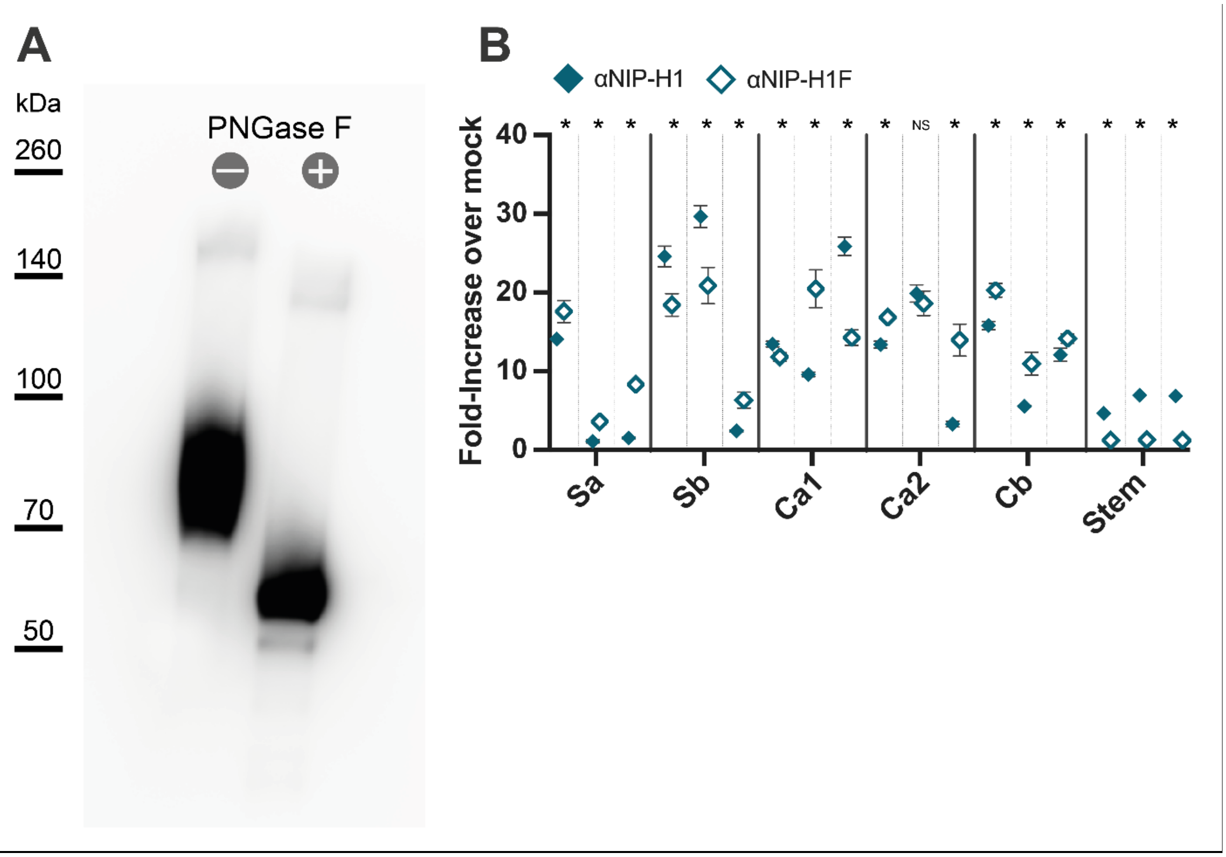

Supplementary Figure 1: HA epitope binding and glycosylation (A) Deglycosylation of recombinant PR8 protein analyzed by Western Blot to elucidate contribution of glycosides on vaccine size. PR8 protein (2.25µg) were treated with PNGase F under denaturing conditions according to the manufacturer's instructions (Cat No. P0704S, New England BioLabs). (**B**) Fold-increase in AUC from ELISA corresponding to Figure 1F. Instead of classifying an epitope as positive based on mean+5×SEM of the mock as in Figure 1F, here the ratio of AUC_{sample} over AUC_{mock} for each mAb in triplicate is calculated and displayed.

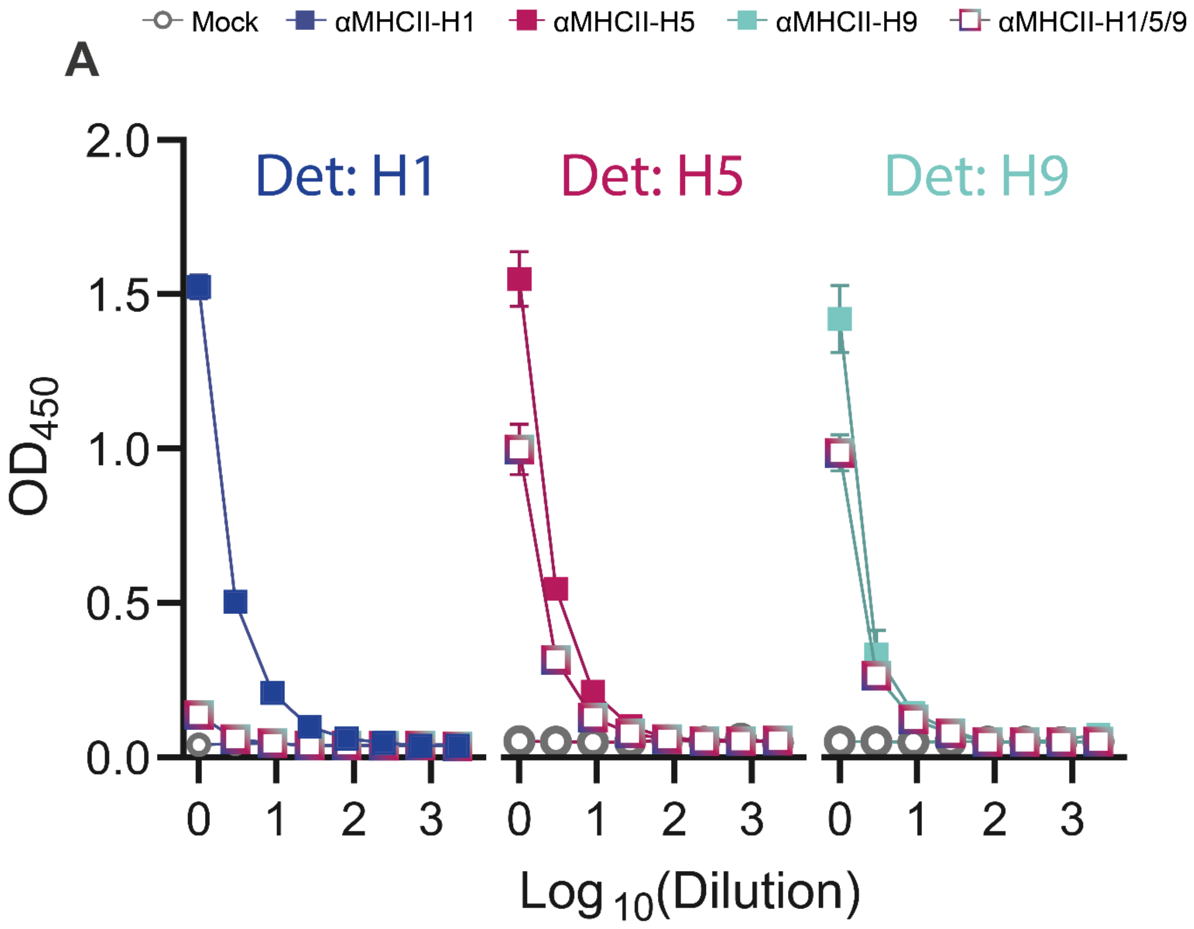

Supplementary Figure 2. In vitro transfection of vaccines encoding influenza H1, H5 and H9. The indicated vaccine plasmids were transiently transfected into HEK293E cells, and vaccine proteins evaluated by ELISA coated with NIP-BSA. Detection was with HA specific mAbs against H1 (clone: H36-4-52), H5 (Cat No. 11048-MM14, Sino Biologicals), or H9 (Cat No. 11229-MM09, Sino Biologicals), as indicated (n=3).

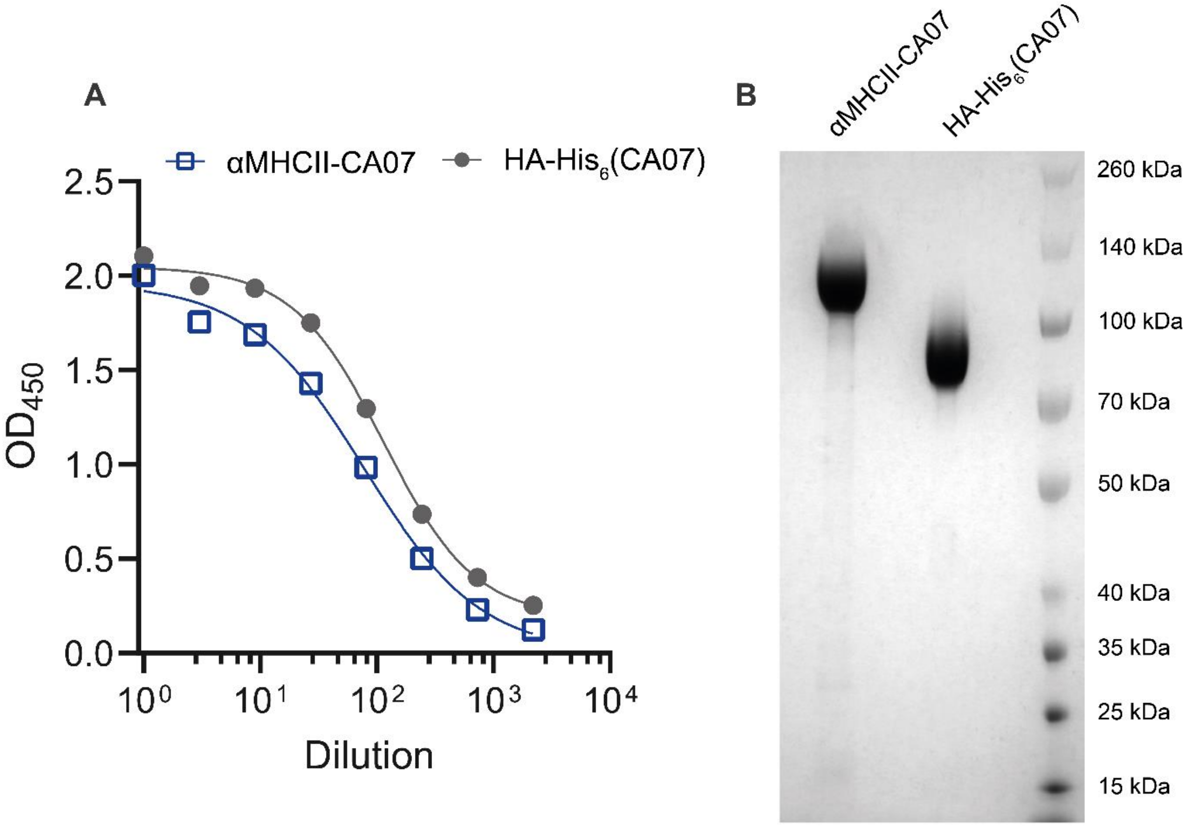

Supplementary Figure 3: **Production of protein vaccine αMHCII-CA07.** HEK293E cells were transfected with plasmids encoding αMHCII-CA07, and the produced proteins affinity purified on an anti-CA07 column (produced in-house, mAb clone: 29E3). (**A)** ELISA analysis of purified αMHCII-CA07 as compared to recombinant HA (CA07) (Cat No. 11085-V08H, Sino Biological). Both samples were loaded in 1 µg/ml in row A, and then serially diluted 3-fold. Capture and detection was performed with the CA07 specific ELISA pair set (Cat No. SEK001, Sino Biologicals). (**B)** SDS-PAGE analysis of purified αMHCII-CA07 as compared to the recombinant protein control from **A**. Proteins were loaded in 5µg on a Bolt™ 4-12% Bis-Tris gel (Cat No. NW04125BOX, Thermo Fisher) under denaturing conditions. Left band: αMHCII-CA07 with a theoretical size of 90 kDa. Right band: control protein with theoretical size of 59 kDa.

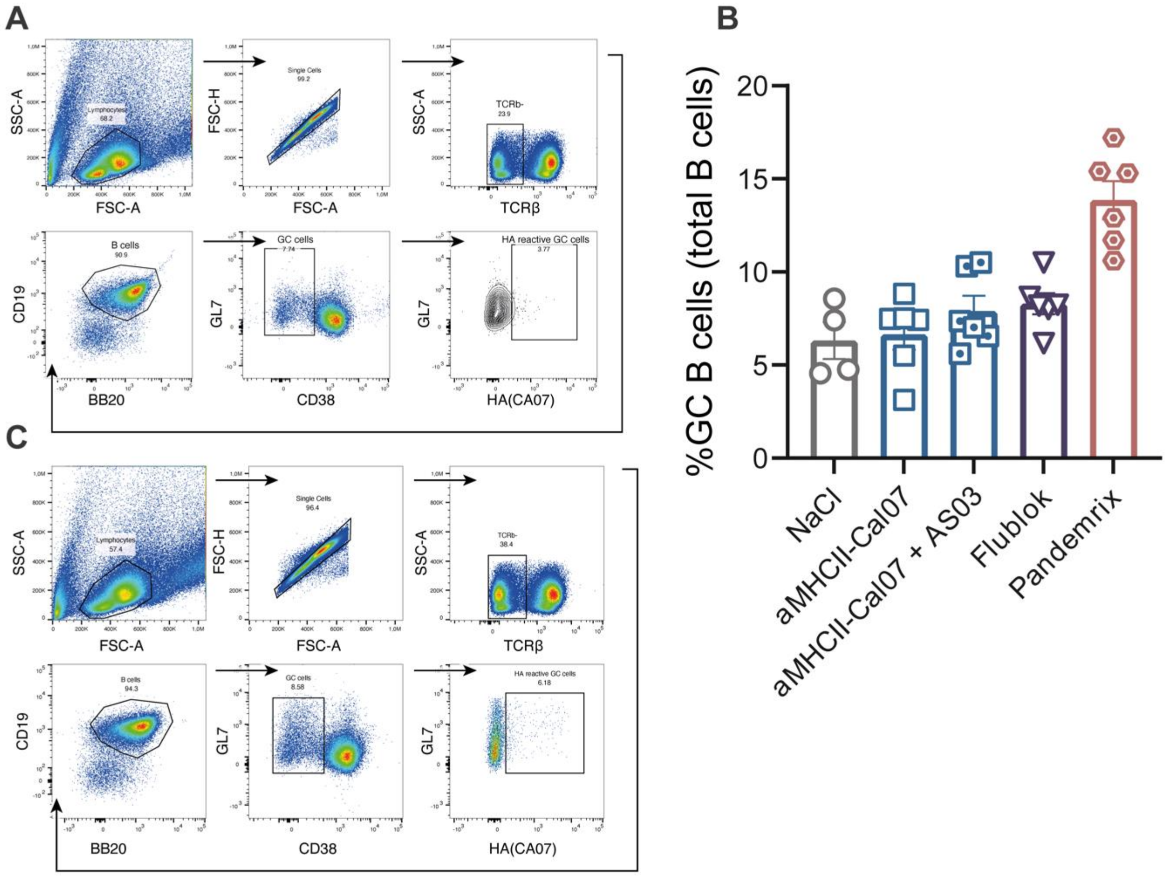

Supplementary Figure 4: **Flow analysis of GC B cells.** Inguinal lymph nodes (draining) were harvested at 14 days post vaccination. (**A)** Representative gating strategy for HA-reactive GC cells with HA-tetramer based staining. Cells were classified as: Single cells, TCR_{β}^{neg}, $CD19^{pos}$, $B220^{pos}$, $CD38^{neg}$, $GL7^{pos}$, HA^{pos}. (**B)** Frequency of GC B cells (% of total B cells) for mice vaccinated with the indicated vaccines, HA-tetramer based staining. (**C)** Representative gating strategy for HA-reactive GC cells with HA-Klickmer® based staining.
